# A Randomized 2x2 Factorial Clinical Trial of Renal Transplantation: Steroid-Free Maintenance Immunosuppression with Calcineurin Inhibitor Withdrawal after Six Months Associates with Improved Renal Function and Reduced Chronic Histopathology

**DOI:** 10.1371/journal.pone.0139247

**Published:** 2015-10-14

**Authors:** R. Brian Stevens, Kirk W. Foster, Clifford D. Miles, Andre C. Kalil, Diana F. Florescu, John P. Sandoz, Theodore H. Rigley, Tamer Malik, Lucile E. Wrenshall

**Affiliations:** 1 Department of Surgery, Wright State University Boonshoft School of Medicine, Dayton, Ohio, United States of America; 2 Department of Pathology and Microbiology, University of Nebraska Medical Center, Omaha, Nebraska, United States of America; 3 Department of Internal Medicine, University of Nebraska Medical Center, Omaha, Nebraska, United States of America; University Hospital Oldenburg, GERMANY

## Abstract

**Introduction:**

The two most significant impediments to renal allograft survival are rejection and the direct nephrotoxicity of the immunosuppressant drugs required to prevent it. Calcineurin inhibitors (CNI), a mainstay of most immunosuppression regimens, are particularly nephrotoxic. Until less toxic antirejection agents become available, the only option is to optimize our use of those at hand.

**Aim:**

To determine whether intensive rabbit anti-thymocyte globulin (rATG) induction followed by CNI withdrawal would individually or combined improve graft function and reduce graft chronic histopathology–surrogates for graft and, therefore, patient survival. As previously reported, a single large rATG dose over 24 hours was well-tolerated and associated with better renal function, fewer infections, and improved patient survival. Here we report testing whether complete CNI discontinuation would improve renal function and decrease graft pathology.

**Methods:**

Between April 20, 2004 and 4-14-2009 we conducted a prospective, randomized, non-blinded renal transplantation trial of two rATG dosing protocols (single dose, 6 mg/kg vs. divided doses, 1.5 mg/kg every other day x 4; target enrollment = 180). Subsequent maintenance immunosuppression consisted of tacrolimus, a CNI, and sirolimus, a mammalian target of rapamycin inhibitor. We report here the outcome of converting patients after six months either to minimized tacrolimus/sirolimus or mycophenolate mofetil/sirolimus. Primary endpoints were graft function and chronic histopathology from protocol kidney biopsies at 12 and 24 months

**Results:**

CNI withdrawal (on-treatment analysis) associated with better graft function (p <0.001) and lower chronic histopathology composite scores in protocol biopsies at 12 (p = 0.003) and 24 (p = 0.013) months, without affecting patient (p = 0.81) or graft (p = 0.93) survival, or rejection rate (p = 0.17).

**Conclusion:**

CNI (tacrolimus) withdrawal at six months may provide a strategy for decreased nephrotoxicity and improved long-term function in steroid-free low immunological risk renal transplant patients.

**Trial Registration:**

ClinicalTrials.gov NCT00556933

## Introduction

Modern transplantation was made possible by anti-rejection therapy that combined steroids with a calcineurin inhibitor (CNI), the class of drugs that includes cyclosporine and tacrolimus (tac). Unfortunately, while largely effective at preventing rejection, these agents also include toxic side effects ranging from pernicious steroid mediated metabolic disturbances to direct CNI nephrotoxicity that contributes to a rate of graft survival that is unacceptable, little better than 50% after ten years.

In clinical trials, CNIs have been minimized, withdrawn, or avoided in hope of reducing graft dysfunction and failure, and possibly cardiac dysfunction, but after prolonged CNI use renal function doesn’t necessarily improve with CNI withdrawal [1–16]. Early replacement of CNI with sirolimus (srl), a mammalian target of rapamycin inhibitor (mTORi, another class of immunosuppressant) may improve renal function [17], but in patients who have progressed to poor function, replacing CNI with sirolimus can result in severe proteinuria and graft failure, suggesting that early discontinuation or minimization of CNI is required to realize significant benefit [18].

To decrease the risk of rejection or donor-specific antibody development when CNIs are minimized early, lymphocyte depletion with rabbit anti-thymocyte globulin (rATG) can be used at transplantation to induce profound immunosuppression [19–28]. Since 1999, we have used induction with rATG to enable early steroid withdrawal (ESW) and minimized maintenance with combined low-dose tacrolimus and sirolimus [29–32]. Sirolimus brings with it significant clinical challenges (e.g., poor wound healing, delayed graft function, hyperglycemia, and proteinuria) [33–38], but its antineoplastic properties are beneficial [39], and the powerfully immunosuppressive sirolimus/tacrolimus combination lowers the risk of rejection after ESW [40,41]. Adverse sirolimus effects can be minimized or avoided by gradual introduction without dose loading; we delay sirolimus for up to six weeks in patients with obesity or poor early renal function [33].

Although historically higher levels of combined tacrolimus/sirolimus have shown reduced graft survival [42], by 2004 our blood-level targets for tacrolimus and sirolimus were 6–8 and 8–12 ng/ml, achievable after lymphocyte depletion with rATG induction (1.5 mg/kg on four alternate days), which facilitates both minimizing and delaying introduction of these maintenance agents. Steroids were administered only during rATG infusion at a total exposure ≤12 mg/kg (maximum ≤1.2 grams). We also collected protocol biopsies to enable early detection and treatment of subclinical pathology [43,44]. With this approach, patient and graft survival equaled outcomes with conventional triple-maintenance therapy (tacrolimus blood level, 8–12 ng/ml; mycophenolate mofetil (MMF), 1 g twice a day; prednisone, 5–20 mg each day) [2,41,45–47]. However, our group and others observed no improvement in renal function.

We hypothesized that more intensive rATG induction could improve renal function by reducing reperfusion injury and safely enabling further mTORi and CNI reduction, or even CNI withdrawal [48–51]. Between 4-20-2004 and 4-14-2009 we conducted a 2x2 factorial trial sequentially comparing single-dose vs. divided-dose rATG induction protocols, and, after six months, aggressive CNI/mTORi minimization vs. delayed CNI withdrawal and replacement with MMF [52]. The 2x2 factorial trial design, although uncommon in transplantation, can address multiple hypotheses much more efficiently with less cost, shorter time frame, and fewer patients [53,54]. In addition, this trial design allows detection of synergistic effects between two treatments.

The primary endpoints of the trial were renal function and chronic graft histopathology. We previously reported that single-dose rATG induction produces better patient survival and graft function with fewer infections. The results reported below show, for the first time, the effects of CNI minimization vs. withdrawal on renal function and chronic histopathology in a prospective randomized trial with ESW.

## Materials and Methods

### Ethics Statement and CONSORT Accounting of Trial

Between 4-20-2004 and 4-14-2009 at the University of Nebraska Medical Center 180 recipients of renal transplants were enrolled and allocated equally into a single center, prospective, randomized, unblinded 2x2 factorial trial of rATG induction and CNI minimization vs. CNI withdrawal. This study was conducted in accordance with the Declaration of Helsinki and Good Clinical Practice guidelines and was approved by the University of Nebraska Institutional Review Board (IRB # 286–03) and registered at ClinicalTrials.gov (#NCT00556933; https://clinicaltrials.gov/ct2/show/NCT00556933). All patients provided written informed consent. The de-identified trial data analyzed for this report (S1 Dataset) and a CONSORT checklist (S1 CONSORT Checklist) are included in the Supporting Information.

### Study Design, Randomization, and Endpoints, and Donor and Recipient Demographics

**Fig 1:**
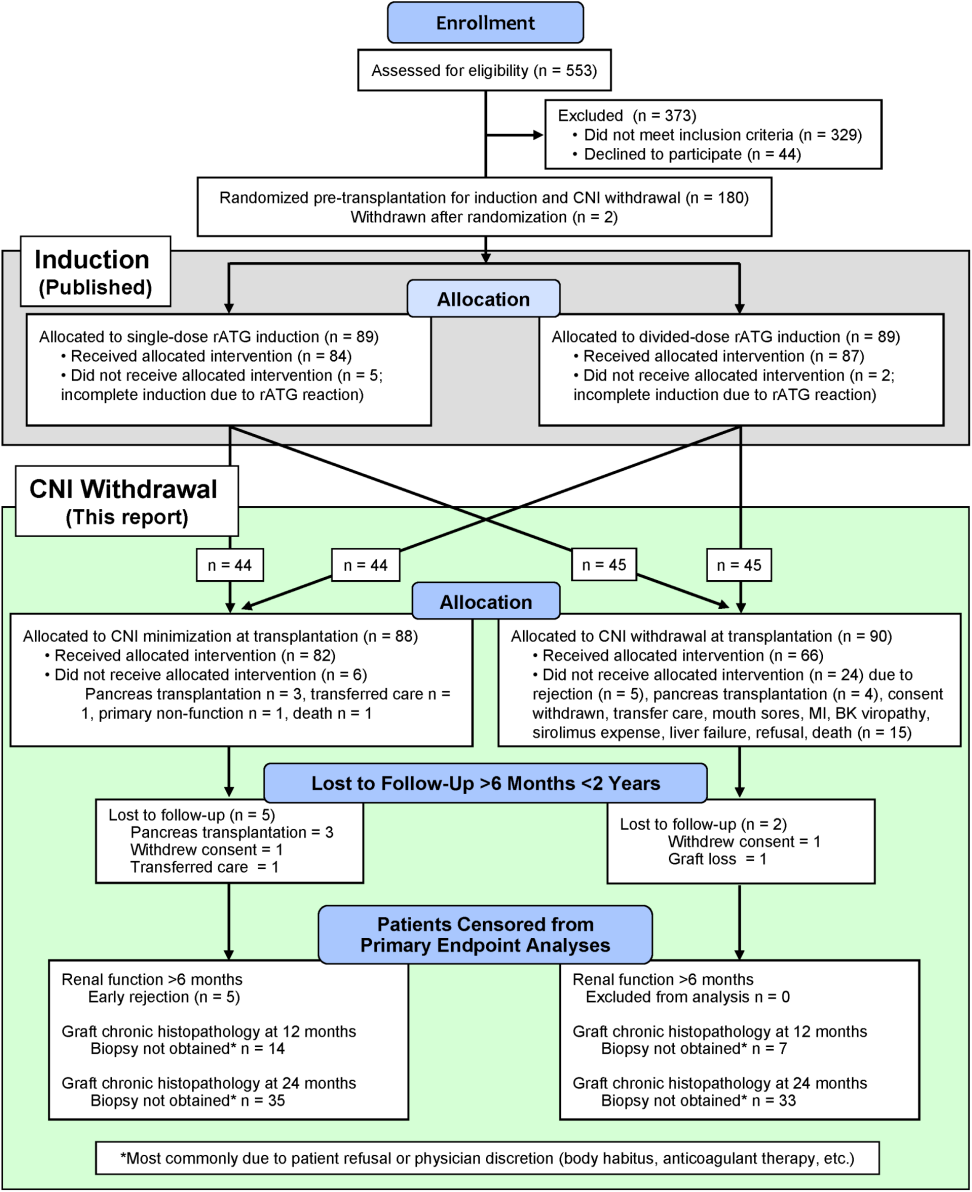
CONSORT flow diagram of randomized 2x2 trial of rATG induction dosing and delayed CNI withdrawal [52]. The study statisticians provided computer-generated randomized assignments in sequentially-numbered, sealed opaque envelopes opened after obtaining consent for trial participation. Randomization included stratification by race (Caucasian/Asian vs. non-Caucasian/Asian), donor type (living vs. deceased), and whether listed for eventual pancreas transplantation. Study patients were identified and enrolled by attending transplant physicians and study coordinators. The integrity of the treatment arm assignments made by this system was monitored and confirmed by the study statisticians. Two patients not meeting enrollment criteria were consented and randomized in error, but were identified before transplantation and removed from participation in the trial.

Primary and selected previous renal transplant recipients (non-immunological causes of graft loss) age >18 were eligible for study participation. Patients were excluded if >65 years, had a panel reactive antibody (PRA) score >75%, were human leukocyte antigen (HLA) identical, or required steroids. Primary endpoints were renal function by calculated glomerular filtration rate (GFR) using the abbreviated Modification of Diet in Renal Disease formula (aMDRD), and acute and chronic renal histopathology (based on Banff ‘05 criteria) [55,56]. A post hoc analysis of graft inflammation, i IFTA (inflammation in areas of kidney interstitial fibrosis and tubular atrophy) and i Total (inflammation throughout the transplanted kidney) was also performed [25,57]. Secondary endpoints included patient survival, graft survival, biopsy-proven rejection, and infectious and non-infectious complications. Details are contained in the Fig 1 legend and in previous publications [52,58]. The study protocol (S1 Protocol), the defining criteria for complications (S1 Criteria) and the treatment protocol for rejection (S1 Treatment) are presented in the online Supporting Information.

### Power Analysis and Statistics

Using a General Linear Model of Repeated Measures, we estimated that 160 patients would provide 80% power with a two-sided 0.05 alpha level to detect a relative 10% difference in calculated GFR after CNI withdrawal during the first year. Enrollment was raised to 180 (+12.5%) during the trial to guarantee sufficient patients undergoing CNI withdrawal.

We compared the effect on renal function (GFR) of CNI withdrawal vs. CNI minimization using a General Linear Model of repeated measures. Banff chronic histopathology scores were compared using Fisher’s Exact Test. Differences in frequencies of infections between groups were tested by Chi-square. Rates of patient survival, graft survival, and rejection were estimated by the Kaplan-Meier method and subjected to Cox proportional hazard analysis. Both donor and recipient factors were analyzed by multivariate logistic regression for possible association with superior renal function. Data are presented as means ± standard deviation unless otherwise specified. PASS, SAS, and SPSS software were used for the power and statistical analyses.

### Immune Suppression

All recipients received 6 mg/kg intravenous rATG (Genzyme Corporation, Cambridge, MA, USA) beginning at transplantation, either a single infusion over 24 hours or four 1.5 mg/kg infusions on alternate days. Except to treat rejection, steroids were administered only with rATG. Treatment for instances of acute tubular necrosis (ATN) or delayed graft function (DGF) was as previously described.

Initial maintenance immunosuppression consisted of sirolimus and tacrolimus. After a screening protocol biopsy at approximately six months, patients randomized to CNI withdrawal were assessed to determine if CNI could safely be replaced with MMF. Protocol biopsies were also obtained at approximately 12 and 24 months.

### Viral, Fungal, and Pneumocystis Prophylaxis

All recipients received oral valganciclovir and clotrimazole for 3 months after transplantation; CMV− recipients with a CMV+ donor received valganciclovir 3 months longer. Patients treated with rATG for rejection received a repeated course of the antiviral prophylaxis used at induction. Pneumocystis prophylaxis was with trimethoprim-sulfamethoxazole or, if allergic to sulfa, diamino-diphenyl sulfone (Dapsone) or aerosolized pentamidine diisethionate for 3 months after transplantation.

### CMV and BK Viral Surveillance

In CMV− recipients transplanted with kidneys from CMV+ donors, blood viral DNA load was checked at 2 weeks, and then monthly for 6 months after valganciclovir was discontinued. Patients with significant leukopenia (white blood cells<2,000) were routinely screened for CMV viremia or infection. All patients underwent urine screening for BK virus at months 1, 3, 6, 9, and 12, and then annually for 5 years. If urine screening showed more than 9 million viral DNA copies, blood was screened also. Patients with renal dysfunction and BK viremia underwent renal biopsy; asymptomatic patients with BK viremia more than 2,000 plasma BK DNA copies per milliliter also were biopsied. This threshold for significant BK viremia is higher than that now used by the authors.

### Calcineurin-inhibitor Withdrawal

Patients were precluded from CNI withdrawal by evidence of sirolimus intolerance or biopsy-proven acute rejection (BPAR) in either a clinically-indicated or six-month protocol biopsy. Twenty-four hours after initiation of MMF therapy (1 g BID), CNI dose was reduced by 50%. Sirolimus dose was increased by 25–50%, then progressively adjusted to achieve the target level of 8–12 ng/ml. CNI was discontinued when sirolimus level reached ≥8 ng/ml.

### Assessment of Renal Function

GFR was estimated with the aMDRD equation, using all blood draws that provided serum creatinine measurements within the specified post-transplantation intervals [55,59,60]. GFR between treatment groups was compared and analyzed using a repeated measures general linear model with maximum likelihood estimation, an approach sensitive to differences between small groups in spite of large intra-patient variance over time. Ordinary least-squares regression was not employed because in the context of repeated measures it can fail to recognize significant effects in the model due to faulty estimation of the covariance structure of the data [61].

### Assessment of Renal Histopathology

We report 12 and 24 month biopsy findings both separately and combined. The combined biopsy score analysis includes a single result from each patient; the most recent biopsy result was used for the 74 patients with biopsies at both times. A post hoc analysis of Banff graft inflammation categories i IFTA and I Total was also performed.

### Diagnosis and Treatment of Acute Rejection

Rejection was confirmed by ultrasound-guided biopsies (BPAR) graded according to Banff 1997 or 2005 criteria [56] and treated as described in S3 (Treatment of Acute Cellular Rejection after Renal Transplantation).

### Assessment of Complications and Infections

Transplant infectious disease specialists (A.K. and D.F.) assessed bacterial and viral infections according to standard criteria.

## Results

### Trial Conduct

#### CONSORT Accounting and Demographics (Fig 1, Tables 1 and 2)

A CONSORT accounting of patients screened for enrollment is presented in Table 1 and flow of patients through the trial is shown in Fig 1. Recipient and donor demographics are shown in Table 2. Follow-up averaged 51.8 ± 15.1 months after transplantation and 45.8 ± 14.9 months after CNI withdrawal. The trial was concluded when all patients had been followed for at least two years.

**Table 1:** CONSORT Accounting of Patients Screened for Study Enrollment.

Potentially eligible patients	604
Not approached	51
Assessed for eligibility	553
Declined participation	44
Did not satisfy study criteria	329 (59%)
Enrolled	180
**Reasons for Study Ineligibility**	
Age >65 years	11%
Requiring steroid therapy	9%
Other medical, social, financial, etc. issues	8%
Age <19 years	7%
History of hepatitis B or C	5%
PRA >75%	3%
Kidney/pancreas simultaneous transplantation	3%
Previous rATG treatment	3%
Previous “other” transplant	3%
History of CMV or BK virus positivity	2%
ABO incompatible or donor-specific antibody	2%
Cardiovascular or vascular issues	2%
Extended criteria donor or donation after cardiac death	1%

**Table 2:** Recipient and Donor Demographics.

CNI Minimization vs. Withdrawal	Minimization	Withdrawal
**# of starting patients**	**88**	**90**
Censored prior to 6 months^1^	5	6
Not withdrawn due to:		
rejection	N/A^3^	5
refused or could not afford sirolimus	N/A	4
PI discretion	N/A	4
BK virus	N/A	2
death	N/A	1
polycystic liver disease	N/A	1
intractable mouth ulcers	N/A	1
myocardial infarction	N/A	1
Censored for rejection ≤6 months	5	N/A
**# patients on treatment**	**78**	**65**
**Recipient Demographics** ITT n =	88	90
Age at transplantation (years)	46.5 ± 11.6	47.9 ± 12.1
BMI	28.2 ± 5.1	28.9 ± 5.5
BMI > 30	33 (38%)	37 (41%)
Males	59 (67%)	62 (69%)
Living donor (related/unrelated)	28 / 25	32 / 23
Deceased donor (DD)	35 (40%)	35 (39%)
DD cold ischemia (hours)	14.5 ± 6.3	14.5 ± 8.2
PRA (%)	0.8 ± 3.8	1.2 ± 7.0
Donor/Recipient height ratio (ITT)	0.98 ± 0.08	1.0 ± 0.07
Donor/Recipient height ratio (On-treatment)	0.98 ± 0.08	1.0 ± 0.07
Antigen mismatch	3.3 ± 1.9	3.5 ± 1.7
CNI withdrawn	N/A	65 (72%)
Race (Non-Caucasian/Asian)	11 (13%)	10 (11%)
CMV serostatus (D+/R- or D+/R+)	54 (61%)	53 (59%)
**Donor Demographics**		
Age at procurement (years)	42.6 ± 12.1	39.3 ± 13.1
Males	41 (47%)	37 (41%)
BMI	27.5 ± 4.9	27.7 ± 4.7
Final creatinine (mg/dl)	1.0 ± 0.5	1.0 ± 0.5
DD grade^2^ A	11 (13%)	9 (10%)
DD grade^2^ B	14 (16%)	12 (13%)
DD grade^2^ C	6 (7%)	8 (9%)
DD <18 years of age	4 (5%)	6 (7%)

^1^Reasons for censoring patients before six months were equally distributed and included withdrawal of consent, scheduled pancreas transplantation, transfer of care, death.

^2^Nyberg deceased donor scoring algorithm [62].

^3^Not Applicable.

#### Total rATG Exposure and Therapeutic Drug Monitoring (Fig 2, Table 3)

**Fig 2:**
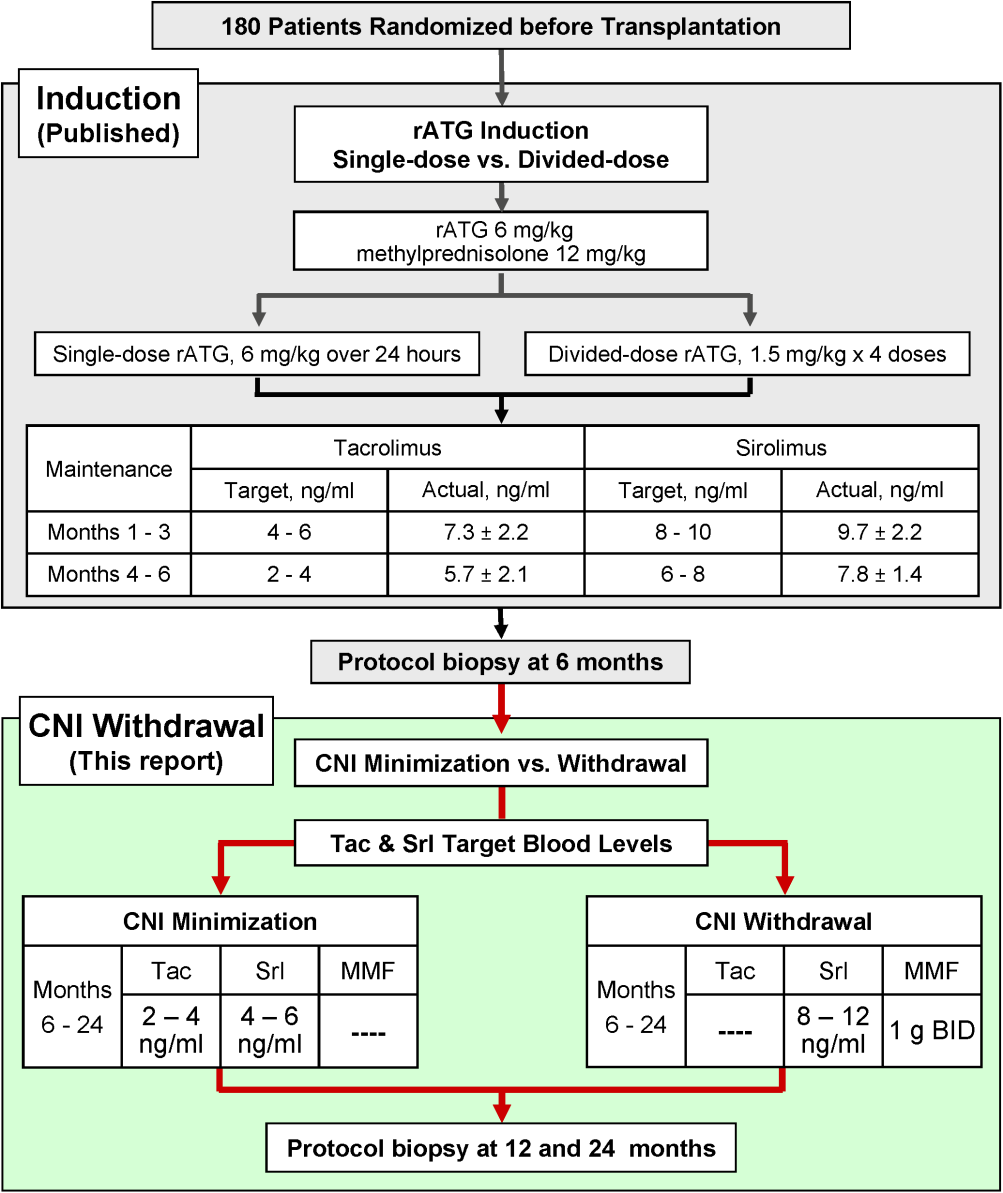
Immunosuppression induction regimens and maintenance target blood levels.

Total rATG exposure (induction, extended rATG for ATN, and as treatment for rejection) between the CNI minimization and withdrawal groups was not different. Respectively, mg/kg rATG were: intent-to-treat, 6.3 ± 1.6 vs. 6.4 ± 1.8 (p = 0.86), and on-treatment, 6.6 ± 2.7 vs. 6.8 ± 2.8 (p = 0.67). Mean immunosuppressant trough levels of both tacrolimus and sirolimus declined over time (in accordance with study design), both during the first six months (Fig 2), and following CNI minimization vs. withdrawal (Table 3). On-treatment blood levels tended to be at or just above the target upper limit except for sirolimus among CNI-withdrawn patients, which averaged in the middle of the target range.

**Table 3:** Targeted and Actual Blood Levels of tacrolimus and sirolimus (ng/ml).

**CNI Minimized Group**							
	Tacrolimus			Sirolimus		Tacrolimus + Sirolimus	
Months	Target		Actual	Target	Actual	Target	Actual
7–12	2–4		5.0 ± 2.0	4–6	6.8 ± 1.6	8–10	11.0 ± 2.4
13–24	2–4		4.6 ± 2.3	4–6	6.6 ± 1.7	8–10	10.5 ± 2.2
**CNI Withdrawn Group**							
	Tacrolimus			Sirolimus		MMF Dose (g, PO, QD)	
Months	Target	Actual		Target	Actual	Actual	
7–12	N/A	N/A		8–12	10.5 ± 1.2	1.9 ± 0.3	
13–24	N/A	N/A		8–12	10.4 ± 1.4	1.9 ± 0.3	

#### Management of CNI Withdrawal

Sixty-six of 90 patients randomized to be withdrawn (73%) met final criteria (Fig 1). An equal number of patients from each rATG induction group underwent CNI withdrawal (single-dose rATG 32 vs. divided-dose rATG 33, p = 1.0). During the first two years post-transplantation, 48 withdrawn patients (74%) remained CNI free. Fourteen patients (22%) returned temporarily to CNI use for an average of 3.4 ± 1.1 months, typically to avoid sirolimus-impaired wound healing after elective surgery. Only three patients (5%) permanently resumed CNI-based maintenance.

#### Steroid Exposure

Of the 178 patients in our trial, 38 (21%) required administration of steroids during the post-transplantation follow-up. Of these, 19 (50%) required a short course (<1month) of prednisone for low-grade rejection. Thirteen of the remaining 19 patients failed this approach or had higher-grade rejection episodes, and were placed on chronic prednisone therapy. Six patients required steroids for unrelated medical reasons (e.g., serum sickness). Of these 19 patients, eleven were randomized to CNI withdrawal and eight to minimization (p = 0.63). Four had undergone CNI withdrawal and one had undergone CNI/sirolimus minimization before initiating chronic steroid therapy for rejection (p = 0.18).

#### No Synergistic interaction between rATG Induction and CNI Withdrawal

No synergistic interaction was observed between single-dose vs. divided-dose rATG induction and later CNI withdrawal in multivariate logistic regression analysis of either renal function or chronic graft histopathology (p >0.40). This validated our 2x2 factorial trial design and justified independent analysis of the effects of CNI withdrawal.

### Primary Endpoints

#### CNI Withdrawal Associates with Improved Renal Function (Fig 3)

**Fig 3:**
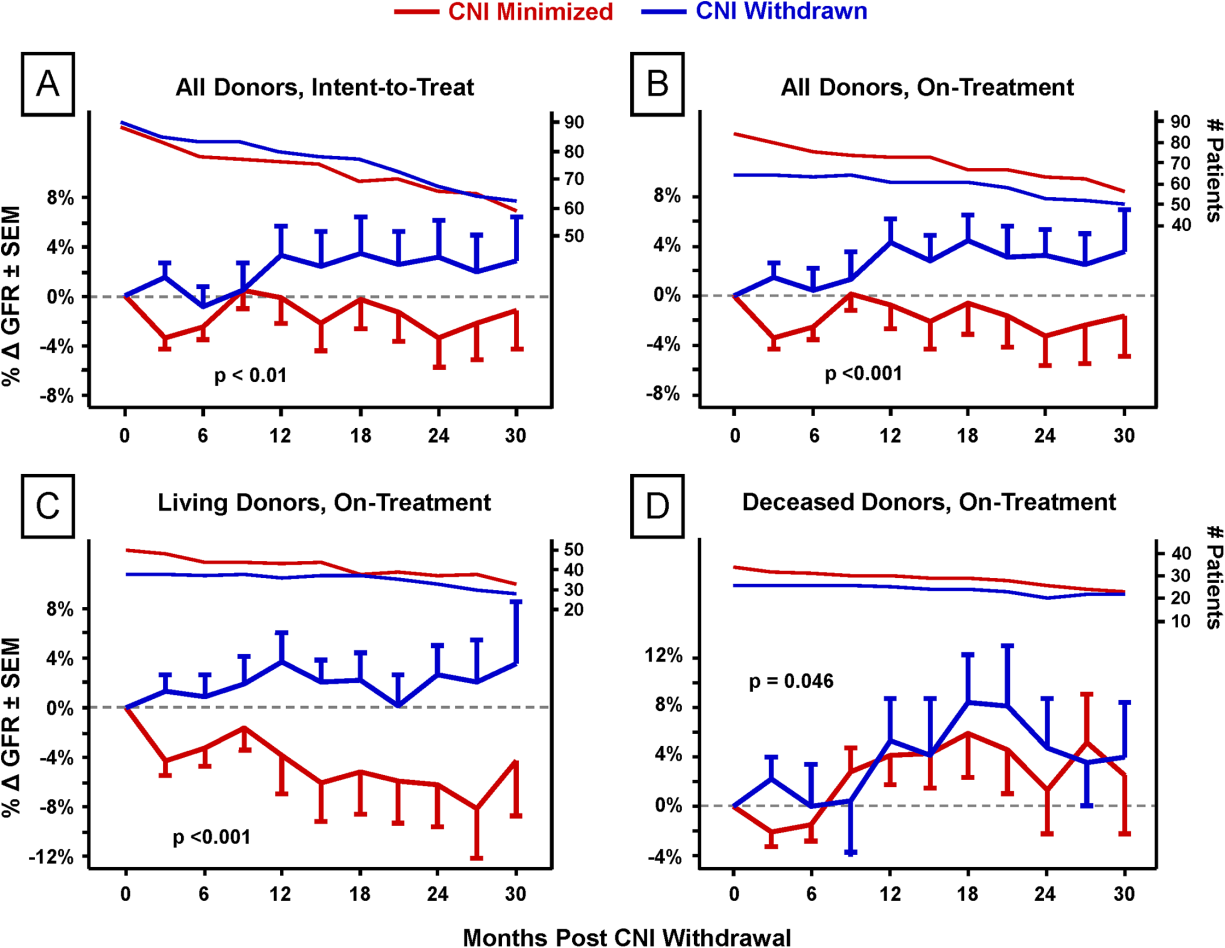
Effect of CNI withdrawal on renal function. The impact of CNI withdrawal on GFR was detected both by intent-to-treat (for all donors, p <0.01) and on-treatment analyses of deviation from a pre-withdrawal baseline GFR for each patient. Post-transplantation months 3–6 GFR values were averaged for use as baselines for CNI-minimization patients; for CNI-withdrawn patients, the three months preceding actual withdrawal were used. To ensure a valid on-treatment comparison of the effects of CNI withdrawal, five patients in the CNI-minimization arm were censored from the analysis due to early rejection (<6 months) because such patients were disqualified from withdrawal in the CNI-withdrawn group. Each time point is the average percentage change of three months of GFR scores.

During the 30 months following CNI withdrawal there was superior renal function among those withdrawn whether analyzed as intent-to-treat (ITT) (p <0.01,) (Fig 3A) or on-treatment (OT) (p <0.001) (Fig 3B). This benefit was most notable among living-donor kidney recipients (p <0.001; deceased donors, p = 0.046; both on-treatment) (Fig 3C and 3D).

In a multivariate logistic regression analysis of CNI minimized (n = 76 ITT, 63 OT) vs. withdrawn (n = 71 ITT, 58 OT) patients that included as variables donor type (p = 0.18), induction group (p = 0.15), donor age (p = 0.69), Nyberg score, and CNI withdrawal, only CNI withdrawal (p = 0.02 on-treatment, p = 0.09 intent-to-treat) and Nyberg score (deceased donors only, p <0.001) associated with renal function improvement after withdrawal (or six months).

#### CNI Withdrawal Associates with Reduced Chronic Renal Histopathology (Figs 4 and 5)

**Fig 4:**
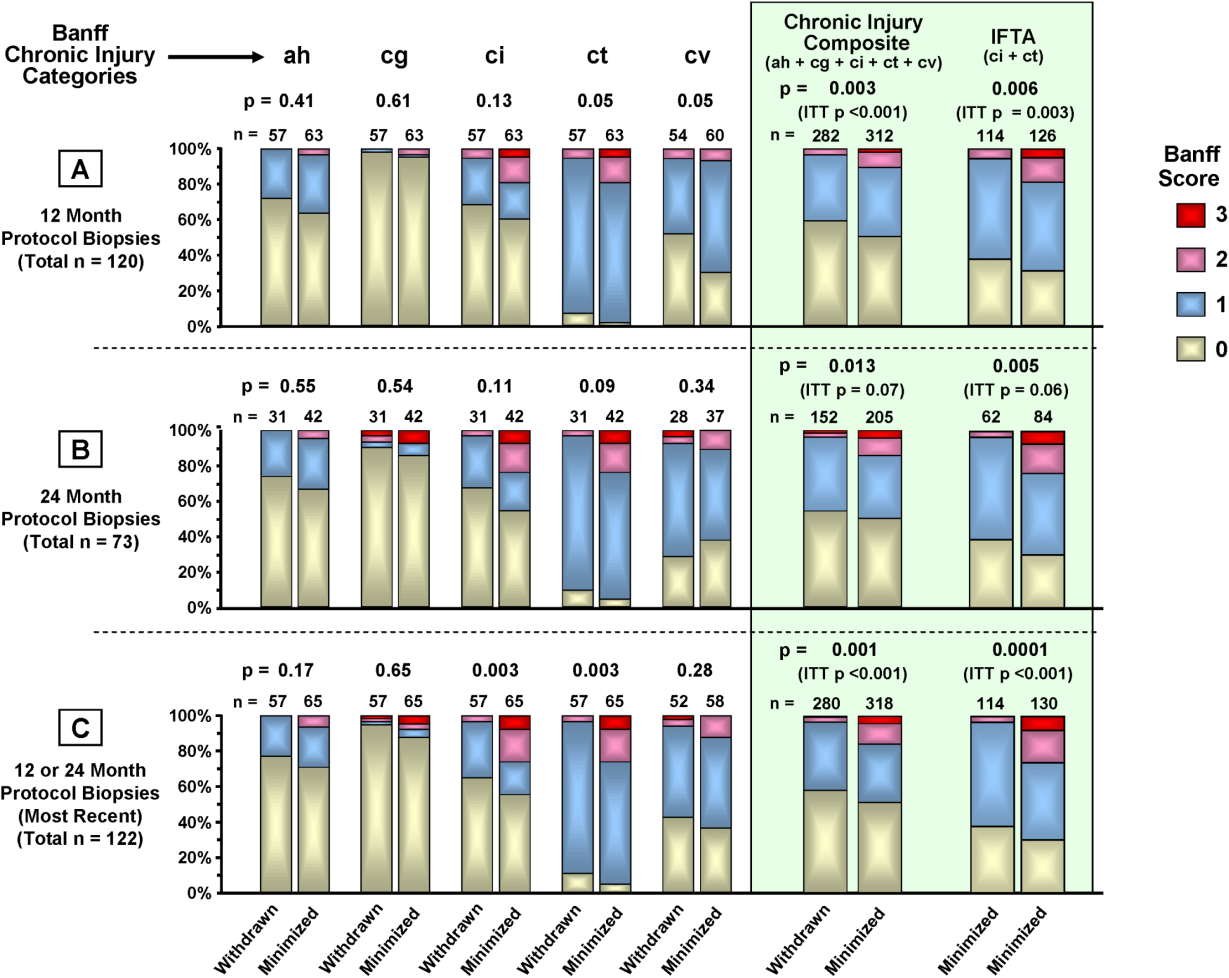
Chronic Banff injury score distributions by on-treatment CNI withdrawal status. Protocol biopsies collected at approximately 12 and 24 months were scored by a transplant renal pathologist blinded to treatment group assignment (author K.F.) for evidence of rejection, BK virus nephropathy, antibody-mediated rejection, recurrent disease, inflammation, and Banff 2005 categories of chronic renal injury. Chronic injury categories were arteriolar hyaline thickening (ah), allograft glomerulopathy (cg), interstitial fibrosis (ci), tubular atrophy (ct), and vascular fibrous intimal thickening (cv). A chronic injury composite score for each group was also created from the five individual injury category scores. Severity scores within each category could be 0 (<5%; none or minimal), 1 (>5%—<25%; mild), 2 (>25%—<50%, moderate), or 3 (>50%, severe). The composite scores shown are the total numbers of ah, cg, ci, ct, and cv scores in each severity grade (0, 1, 2, or 3). The proportions of patients in each severity grade (0, 1, 2, and 3) for both the individual categories and the composite were compared using Fisher’s exact test. Intent-to-treat (ITT) p-values are included in the figure.

217 protocol biopsies were obtained from 138 patients (78% of enrollment) at 12 (Fig 4A) or 24 months (Fig 4B); 79 (57%) of these patients were biopsied at both times. For patients biopsied at both times, the most recent biopsy was used for the 12/24 month composite analysis (Fig 4C). For intent-to-treat analysis, 71 of 90 (79%) CNI-minimized and 67 of 88 (76%) CNI-withdrawn patients were biopsied. For on-treatment analysis, 57 of 65 (88%) CNI-withdrawn patients underwent protocol biopsy at either 12 or 24 months, or both. 67 of 83 (81%) CNI-minimized patients underwent protocol biopsy at either or both 12 or 24 months. Five patients in the CNI-minimization arm were censored from the analysis due to early rejection. 48% of on-treatment CNI-withdrawn patients and 50% of on-treatment CNI-minimized patients were biopsied at 24 months.

**Fig 5:**
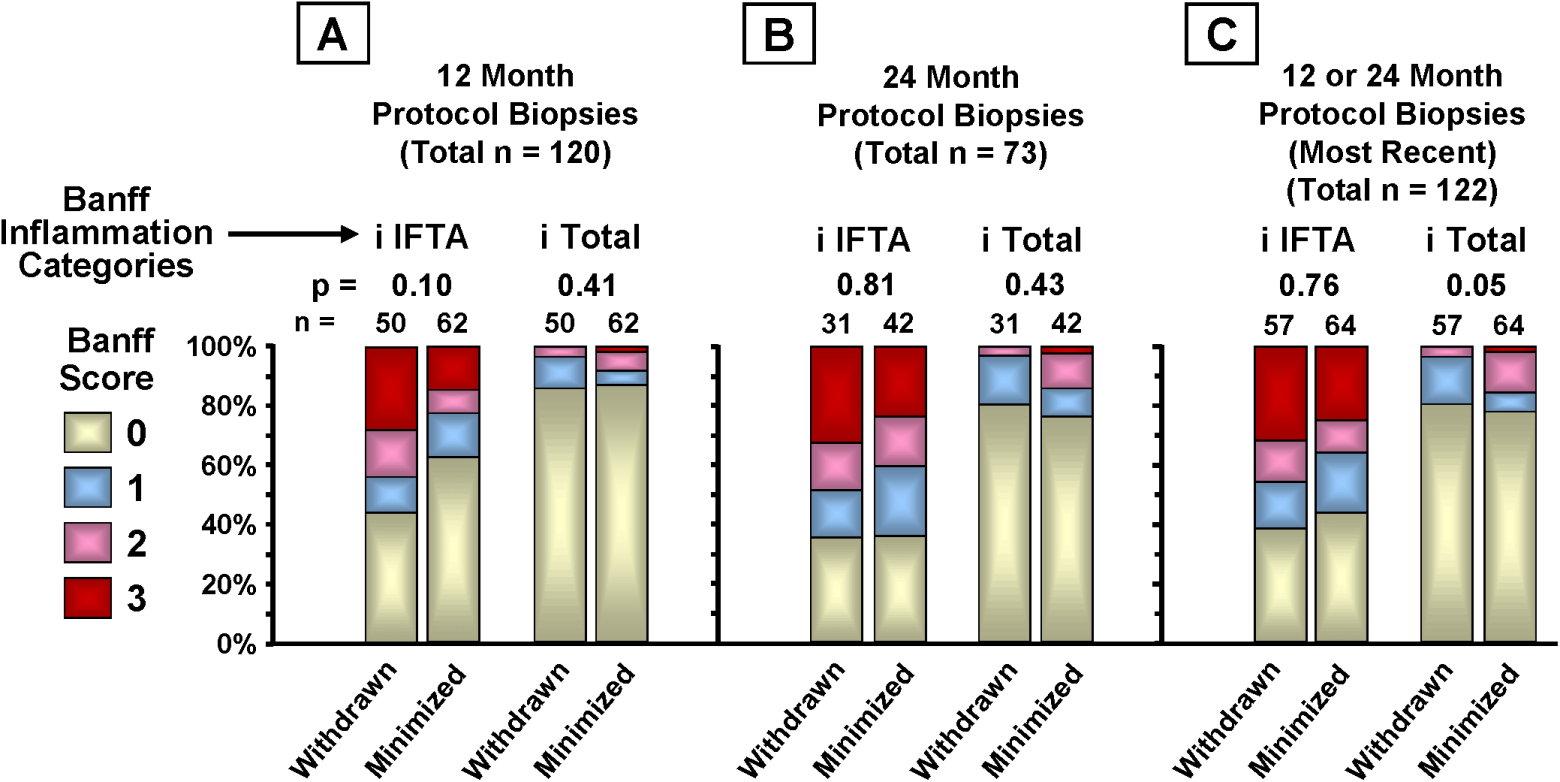
Post hoc on-treatment analysis of graft inflammation (i IFTA & i Total). Inflammation was scored both within areas of interstitial fibrosis and tubular atrophy (i IFTA) and throughout the biopsy (i Total). Severity scores within each category could be 0 (<5%; none or minimal), 1 (>5%—<25%; mild), 2 (>25%—<50%, moderate), or 3 (>50%, severe). The proportions of patients in each score category (0, 1, 2, and 3) were compared using Fisher’s exact test.

There was significantly less chronic injury in the on-treatment CNI withdrawal group in the composite scores, primarily due to less IFTA (ci and ct). CNI withdrawal associated with numerically lower chronic histopathology scores in both the 12 and 24 month protocol biopsies except for cv at 24 months. Multivariate logistic regression analysis showed some measures of donor organ quality associated with elevated Banff chronic histopathology scores; donor type for categories ah (p = 0.04) and ci (p = 0.05), donor age for ah (p = 0.07) and deceased-donor Nyberg score for ci (p = 0.01). Nyberg score also associated significantly with a chronic injury composite of ah + ci + ct + i total (p = 0.01).

The early trend toward more i IFTA among CNI withdrawn patients at 12 months (p = 0.10) (Fig 5A) was absent by 24 months (p = 0.81) (Fig 5B) and in the combined 12 and 24 month biopsies (p = 0.76) (Fig 5C). The combined biopsies also showed lower i Total scores among the CNI-withdrawn group (p = 0.05) (Fig 5C).

### Secondary Endpoints

#### CNI Withdrawal Did Not Affect Patient and Graft Survival

**Fig 6:**
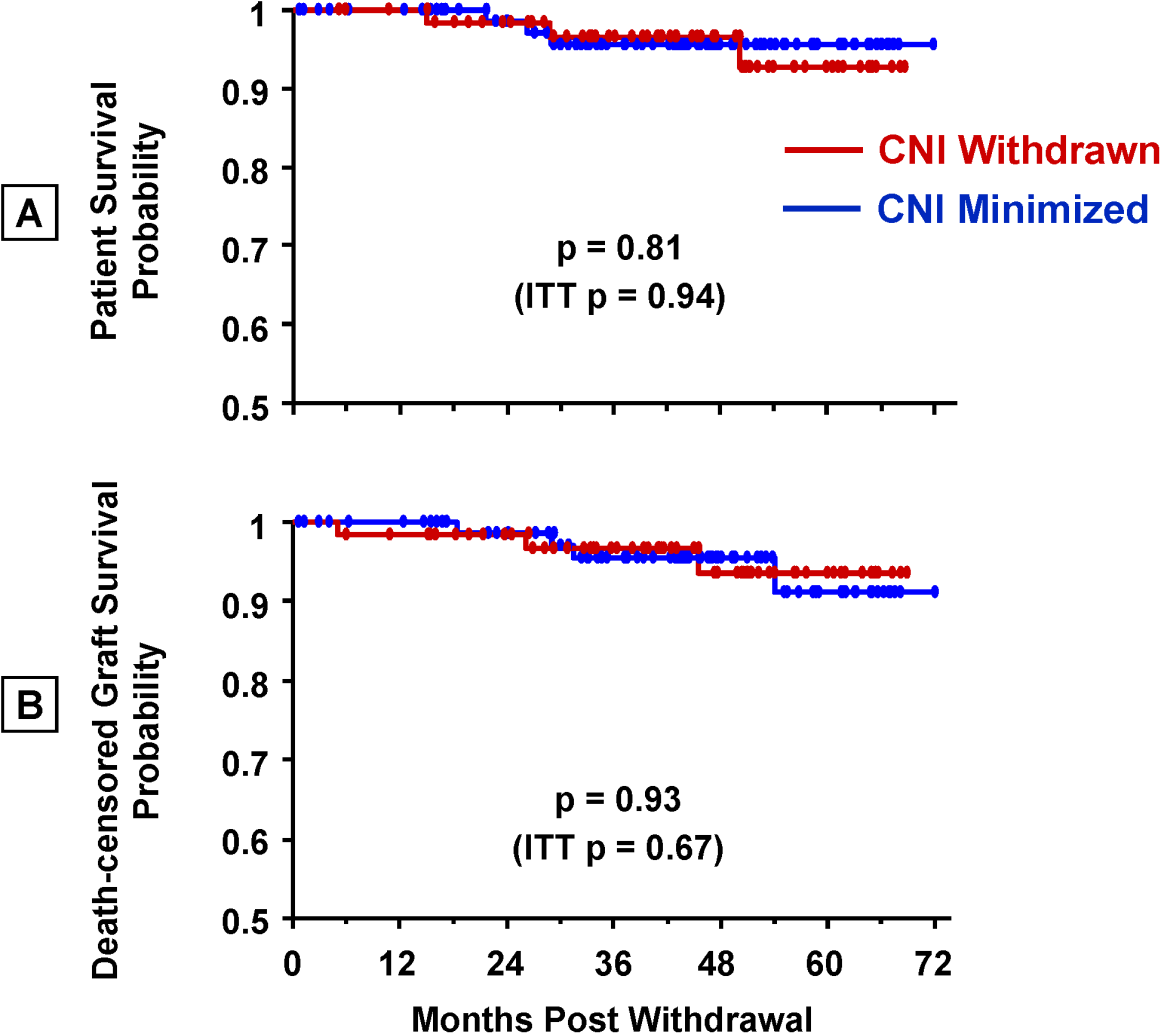
On-treatment patient and death-censored graft survival after CNI withdrawal. P-values for the intent-to-treat analyses are also shown.

For over six years after transplantation there were no apparent differences in patient survival (Fig 6A) or death-censored graft survival (Fig 6B) between the CNI withdrawal and minimization groups.Eight patients died during the course of study follow-up; causes of the five deaths in the CNI-minimized group included MI (1), cancer (3), and sepsis (1). Patients in the CNI-withdrawn group died of bacterial sepsis (1), pulmonary embolism (1), and an accidental drug overdose (1). Deaths occurred at an average of 2.3 ± 1.4 years after transplantation, at patient ages 59.2 ± 2.9 years (CNI minimized) and 52.0 ± 11.7 years (CNI withdrawn).

There were seven instances of death-censored graft loss during follow-up. Four grafts were lost in the CNI-minimized group due to: humoral rejection/BK nephropathy (1), chronic/acute rejection (2), and non-compliance (1). Three grafts were lost in the CNI-withdrawn group due to: transplant glomerulopathy (1), chronic/acute rejection (1), and non-compliance (1).

#### CNI Withdrawal Did Not Increase Risk of Acute Rejection

Protocol biopsies at 12 and 24 months (Table 4) showed no significant differences in frequency or severity of rejection between CNI groups. There was one finding of BK virus at 12 months in a CNI-minimized patient and one at 24 months in a CNI-withdrawn patient. We found no evidence of grade 2 or 3 rejection, antibody-mediated rejection, or recurrent disease in any protocol biopsy.

**Table 4:** Rejection in Protocol Biopsies.

		Banff Rejection Grade		
Time after Transplantation	CNI Regimen	Borderline/ Suspicious	1A	1B
12 ± 2 Months	Minimized n = 69 (72%)	6 (9%)	1 (1.4%)	1 (1.4%)
12 ± 2 Months	Withdrawn n = 63 (77%)	8 (13%)	5 (8%)	1 (1.6%)
24 ± 4 Months	Minimized n = 46 (52%)	8 (17%)	1 (2.1%)	0
24 ± 4 Months	Withdrawn n = 34 (52%)	5 (15%)	0	0

**Fig 7:**
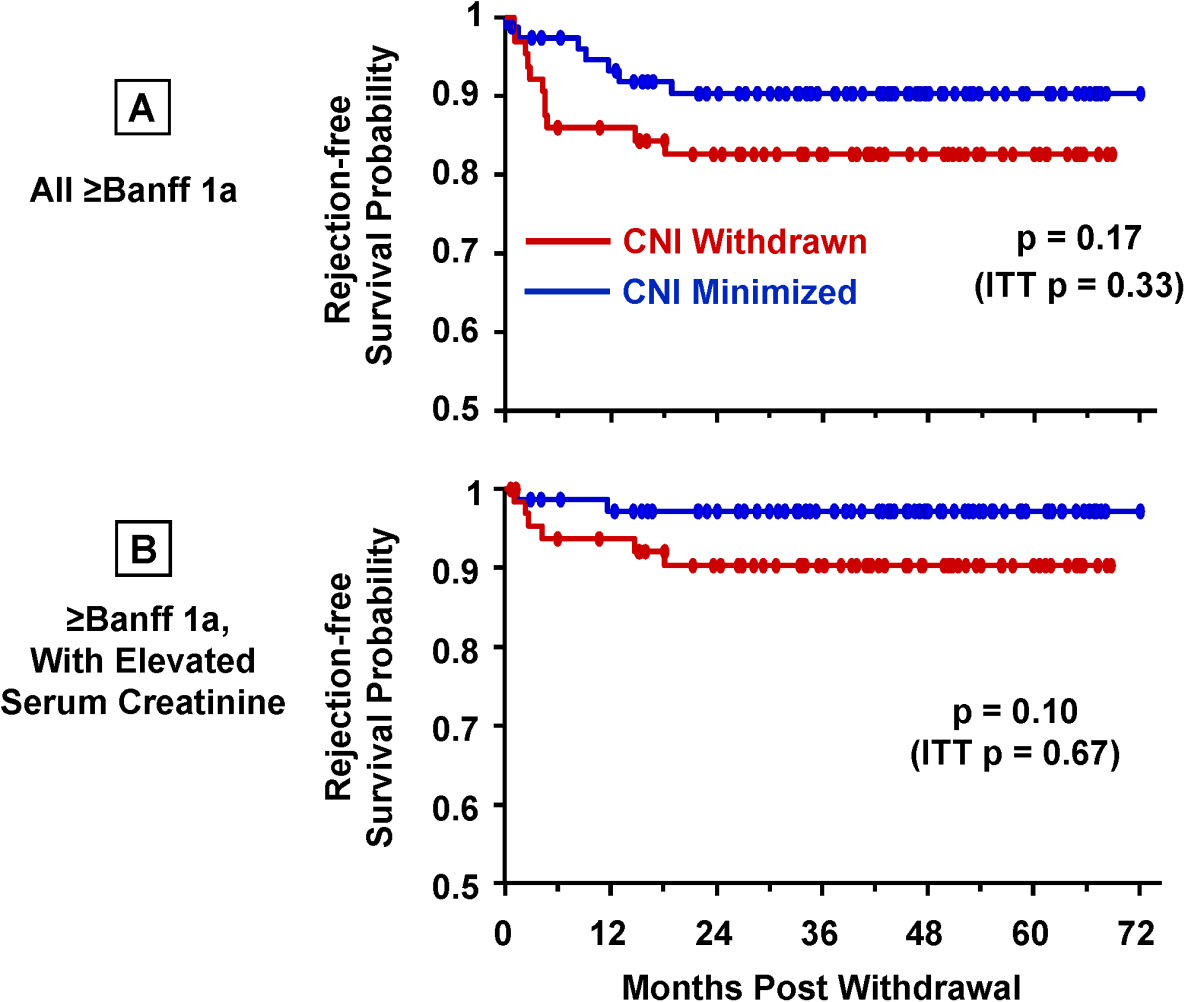
Rejection after CNI withdrawal. On-treatment Kaplan-Meier analyses and log-rank tests (CNI minimized, n = 77; withdrawn, n = 64); intent-to-treat (ITT) p-values are included in the figures. (A) All biopsy findings of rejection ≥Grade 1A. The majority of rejection episodes after six months were low-grade Banff 1A: CNI minimized, 73% (4/7); CNI withdrawn, 70% (7/10). The number of higher-grade rejection episodes was similar between the two groups: CNI minimized, 27% (3-IB); CNI withdrawn, 30% (2-IB, 1-IIA). (B) Rejection ≥Grade 1A accompanied by clinically elevated serum creatinine (>30% above baseline). Only 18% of all rejection events in the CNI-minimized group were associated with an increase in serum creatinine. In contrast, 60% of rejection events in the CNI-withdrawn group associated with elevated serum creatinine (p = 0.10).

In the intent-to-treat analysis beginning at CNI withdrawal (or six months for those not randomized to CNI withdrawal), the risk of rejection for the CNI-withdrawn vs. minimized group, whether or not associated with graft dysfunction, did not approach statistical significance (p = 0.67 and 0.33, respectively). Because 28% of patients randomized were unable or unwilling to undergo CNI withdrawal, we performed an on-treatment analysis of rejection events after CNI withdrawal (or six months) (Fig 7A and 7B). There was a trend toward more rejection after CNI withdrawal (p = 0.17; Fig 7A) which became more pronounced among patients who also exhibited graft dysfunction (p = 0.10; Fig 7B). The majority of on-treatment rejection events in the CNI withdrawal group (82%) occurred within six months of withdrawal, compared with 29% in the CNI-minimized group during the same time (p = 0.049).

Multivariate logistic regression analysis of covariates likely to associate with rejection after withdrawal (single-dose rATG induction, deceased donor, CIT, gender, age, and sirolimus levels) showed an increased risk of rejection with increasing CIT (p = 0.05), and a trend with younger recipient age (p = 0.09). Rejection was not significantly associated with deceased donors (p = 0.15), single-dose rATG induction (p = 0.19), or average sirolimus levels after withdrawal (p = 0.72).

#### Complications and Infections after CNI Withdrawal

There were no significant differences in rates of infectious or non-infectious complications between the CNI-withdrawn and CNI-minimized groups in either the intent-to-treat or on-treatment analyses. However, among the CNI-withdrawn, there were trends in the on-treatment analysis toward a greater frequency of transplant wound hernias requiring repair and fewer instances of ureteral obstruction (Table 5).

**Table 5:** Complications and Infections.

Intent-to-treat		On-treatment	
**Complications**	**CNI Minimized**	**CNI Withdrawn**	**p-value**	**CNI Withdrawn**	**p-value**
n =	88	90		65	
rATG reaction	3 (3.4%)	6 (6.7%)	0.5	2 (5%)	0.70
ATN/DGF	4 (4.5%)	8 (8.9%)	0.37	6 (9%)	0.32
Myocardial infarction	1 (1.1%)	2 (2.2%)	1.0	1 (2%)	1.0
Cardiac arrhythmia	0 (0%)	2 (2.2%)	0.50	2 (3%)	0.18
Serum sickness	1 (1.1%)	2 (2.2%)	1.0	2 (3%)	0.57
Lymphocele requiring drainage	4 (4.5%)	6 (6.7%)	0.75	5 (8%)	0.50
Wound complications	2 (2.3%)	3 (3.3%)	1.0	2 (3%)	1.0
Transplant wound hernia requiring repair	7 (8.0%)	13 (14.4%)	0.24	11 (17%)	0.12
Ureteral obstruction	5 (5.7%)	2 (2.2%)	0.28	0 (0%)	0.07
Cancer	4 (4.5%)	5 (5.6%)	1.0	3 (5%)	1.0
**Infections**	**CNI Minimized**	**CNI Withdrawn**	**p-value**	**CNI Withdrawn**	**p-value**
n =	88	90	–	64	–
Urinary tract infection	17	18	1.0	13	1.0
Bacterial pneumonia	5	3	0.50	1	0.40
Bacteremia	7	5	0.56	4	0.76
Fungal	1	2	1.0	1	1.0
CMV infection (disease, viremia, syndrome)	3	6	0.50	3	0.70
BK nephropathy	3	3	1.0	1	0.64
PTLD	3	0	0.12	0	0.26
**Total infections**	**39**	**37**	**0.76**	**23**	**0.32**

#### Combined Impact of Single-dose rATG Induction with CNI Withdrawal (post hoc analysis) (Fig 8)

**Fig 8:**
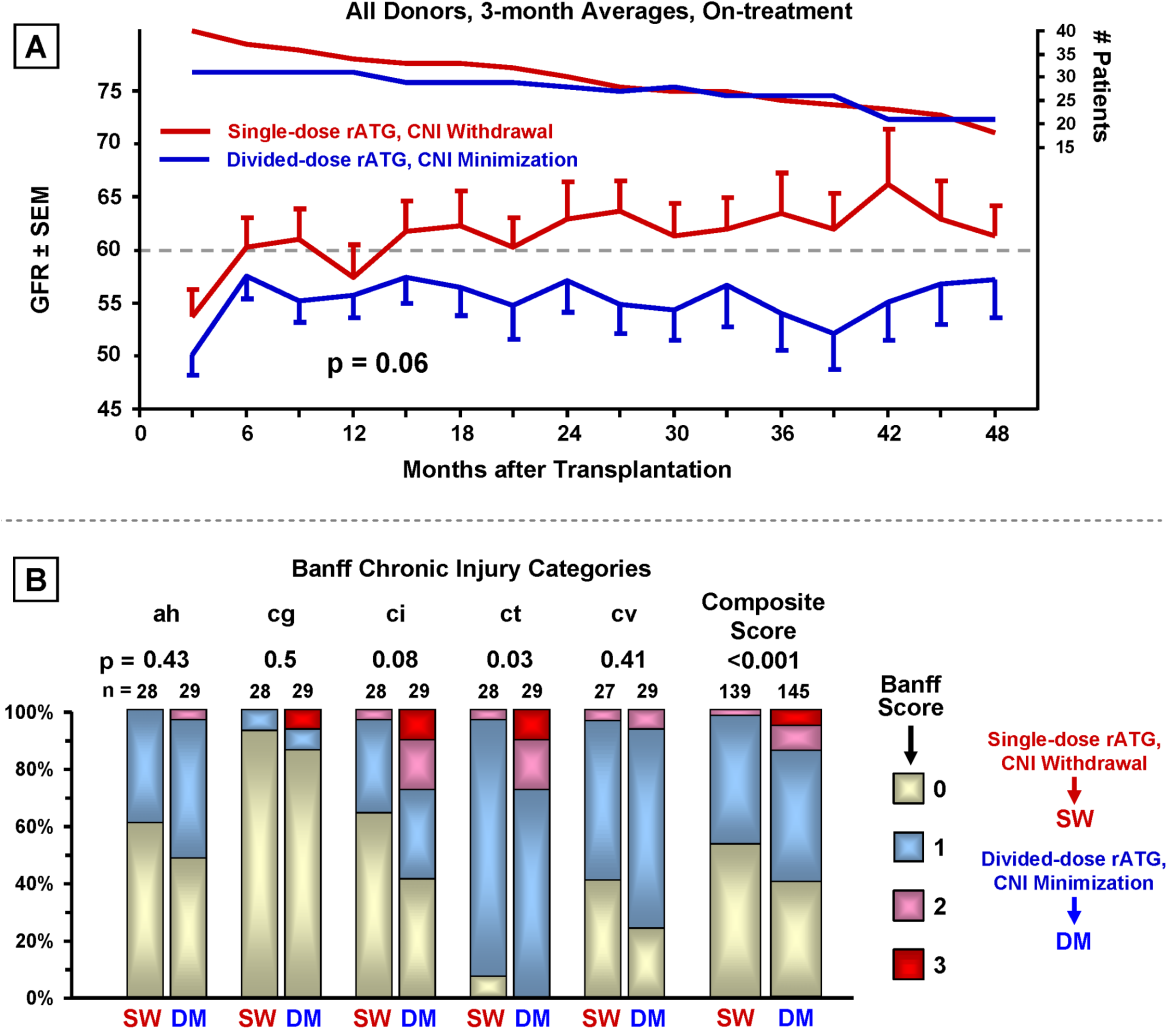
Combined impact of single-dose rATG induction with CNI withdrawal. On-treatment comparison of best and worst treatment combinations (single-dose rATG with delayed CNI withdrawal vs. divided-dose rATG with delayed CNI minimization). (A) Renal function analyzed as in Fig 3. (B) Chronic Banff renal graft histopathology as in Fig 4.

Superior renal function (p = 0.06) (Fig 8A) and significantly less chronic injury in the composited Banff categories (p <0.001) (Fig 8B) was observed in patients who received the combination of single-dose rATG and CNI withdrawal vs. divided-dose rATG with CNI minimization.

## Discussion

The trial presented here would be challenging to perform with a multi-center design (multiple protocol biopsies, maintenance of immunosuppression agent blood levels within narrow ranges, etc.). Although multi-center trials are the gold standard, a single-center study offers more consistent long-term follow-up and tighter control over multiple parameters, thus enabling the implementation of a complex protocol. Our single-center trial benefited from long-term patient oversight according to standardized written protocols and from the 2x2 design, which efficiently and cost-effectively allowed testing two interventions.

Combining the two induction groups for analysis of the effects of CNI withdrawal vs. minimization is appropriate when it can be shown there is not a significant interaction between the induction treatment and the subsequent CNI maintenance regimen used. If a significant interaction between the induction and CNI maintenance regimens were observed, a four-group analysis would have been appropriate, and indeed, is the only option allowed in reporting our trial outcomes at clinicaltrials.gov. However, the interaction of rATG induction regimen and CNI withdrawal was not statistically significant, thereby qualifying induction and withdrawal for analyzing and reporting separately.

We have previously shown that single-dose rATG induction has a reduced complication profile with superior long-term graft function in recipients of deceased donors after ESW and CNI minimization [52,58]. Although not powered specifically to evaluate patient survival and infection rates, use of single-dose rATG resulted in fewer infectious complications and improved patient survival. The difference in patient survival was unexpected, but divided-dose rATG associated with more severe and prolonged CD4 leukopenia, which correlated with multiple and more severe infections. Cox proportional hazard modeling showed that an increased hazard of mortality significantly associated with divided-dose rATG, lower lymphocyte count, serious infection, and deceased donors. Mortality was increased 27-fold among patients with all four conditions [52].

The results reported here show, for the first time, that CNI withdrawal (vs. CNI/sirolimus minimization) in a steroid-free environment improves renal function and reduces chronic allograft injury without significant difference in patient or death-censored graft survival. As mentioned above, the trial outcomes are reported at the clinicaltrials.gov website as a comparison of four (instead of two) groups, where even these reduced group sizes show a significant chronic renal histopathology benefit to single-dose rATG induction followed by CNI withdrawal (p = 0.0004).

The analysis of the repeated measures/longitudinal data in this study was carried out with linear mixed models for longitudinal data. These models use maximum likelihood estimation in which all available data, not just complete cases, are used in the analysis [61]. With the advent of powerful desktop computers and more sophisticated statistical software there is no need to compromise data by imputing missing values, and data need not be collected only at rigidly pre-specified time points. Clinical research need not be restrained by the computational limitations of the past. In this trial we benefitted greatly by being easily able to model for different co-factors, e.g., induction regimen, withdrawal status, CNI levels, sirolimus levels, donor type, donor quality, age, race, gender, etc. With this approach we were able to show that what associated with the primary endpoints (renal function and chronic histopathology composite scores after CNI withdrawal) was CNI withdrawal status.

Overall, the safety of CNI withdrawal and minimization appeared similar, with no differences in rates of infectious complications or malignancies. Among the CNI-withdrawal group in the on-treatment analysis, there was a suggestion of an increased rate of transplant wound hernia repair and fewer instances of ureteral obstruction, probably related, respectively, to increased sirolimus exposure and the exclusion from CNI withdrawal of patients with early ureteral complications.

Prospectively randomized multi-center trials addressing the possible benefit of CNI withdrawal have reported frequent adverse sirolimus effects and sirolimus discontinuation, likely due to high target levels [63]. In contrast, our trial’s comparator arm minimized CNI and sirolimus after ESW. We use low-dose sirolimus without loading; only 5%-10% of our patients discontinue sirolimus because of complications, compared to up to 32% in studies targeting higher levels [33,38].

As with our study, discontinuing CNIs in the Spare-the-Nephron trial associated with improved renal function (at 12 months) without increased BPAR or graft failure [64]. A direct comparison is difficult, as neither induction regimen nor steroid maintenance were specified in the trial protocol. In contrast to our study, renal function was not different at 24 months, however at that point only half of their patients were on-treatment. An on-treatment analysis was not reported.

Lebranchu et al, using non-lytic induction in a multi-center trial, sequentially withdrew CNIs (three months) and steroids (eight months) [18,65]. At 10 months, their BPAR rates were 17% (sirolimus+MMF) and 8% (CSA+MMF). In our on-treatment analysis at 12 months, rejection rates were 6% (CNI withdrawn) and 3% (CNI minimized) (p = 0.34). The lower rates of BPAR in our trial are likely due to our use of rATG induction along with steroid withdrawal within seven days [66,67]. After 72 months, our cumulative rejection rates were 18% (CNI withdrawn) and 10% (CNI minimized) (p = 0.17). Comparing these two studies suggests that the risk of rejection in association with CNI withdrawal can be minimized with the use of lytic induction. In addition, there are benefits to single-dose rATG induction not realizable with either divided-dose rATG or non-lytic induction [48,49,68,69].

In the BENEFIT belatacept registry trial, CNI avoidance (vs. maintenance) produced approximately 6% higher GFR levels at three years [70,71]. We observed an improvement in GFR of 4% at three years; the greater improvement in the BENEFIT trial likely reflects higher CNI exposure in their control arm when the relative pharmaceutical potency of the CNI used is accounted for (CSA ~150 ng/ml vs. our trial, ~5 ng/ml tacrolimus) [72]. The BENEFIT/EXT phase III trial (extended-criteria donors) also showed better renal function with CNI avoidance and maintenance with MMF and prednisone [73]. Belatacept appears to allow for both early steroid withdrawal and CNI avoidance when used in combination with lytic induction [74,75]. In our trial, patients with higher Nyberg score donors showed no renal function improvement after CNI withdrawal, suggesting that regimens avoiding both sirolimus and CNI may be better for such patients.

Because protocol biopsies are typically not performed in multicenter trials, there are fewer comparisons in this area. Protocol biopsies were performed at 12 months in the BENEFIT trial, however, which showed a significantly lower prevalence and severity of chronic allograft nephropathy in association with CNI avoidance. Similarly, we found a significant reduction at both 12 and 24 months in the Banff chronic allograft injury composite score and IFTA. Nankivell reported rapid development of chronic allograft nephropathy in kidney/pancreas recipients (>95% CAN-1 by year two) [7,76]. Despite a comparatively slower rate of chronic injury development in our study, we still observed significantly less chronic histopathology among the CNI-withdrawn patients, independent of induction regimen.

Among CNI-withdrawn patients there was a trend toward more inflammation associated with IFTA at 12 months (p = 0.10), possibly associated with the trend toward increased rejection soon after withdrawal. However, this trend disappeared with subsequent biopsies. While there was less total graft inflammation (i Total) in the CNI withdrawal group at both 12 and 24 months, the difference only became significant in the combined 12 and 24 month analysis (Fig 3B). Others have shown that i IFTA and i Total are predictors of reduced graft function and long-term survival [25,57]. The decrease in chronic allograft injury plus stable/reduced inflammation associated with CNI withdrawal suggests that the slight increased risk of low-grade rejection is an acceptable trade-off, but underscores the value of protocol biopsy in the early detection and amelioration of injury processes [9,43,77,78].

The combined results from our 2x2 trial support the notions that single-dose rATG induction improves renal function and safety and CNI withdrawal significantly improves long-term renal function and reduces renal histopathology. Single-dose rATG induction followed by delayed CNI withdrawal appears to have the greatest impact on renal function and chronic histopathology. We believe that with close graft monitoring after CNI withdrawal, and by returning those with rejection to CNIs, there is unlikely to be an increased risk of graft loss.

The trial’s limitations include being a non-blinded single-center trial with limited racial diversity, a predominance of living donors, and a high percentage of patients not undergoing CNI withdrawal. 27% of patients randomized to CNI withdrawal were not able to be withdrawn (primarily due to rejection), but patients were withdrawn in sufficient numbers to enable detection of a significant effect on both renal function and chronic graft histopathology. As mentioned above, this is likely due to the sensitivity resulting from using all available data in the General Linear Model used to analyze renal function. Although we must acknowledge the possibility of a false-positive finding, the improved renal function after CNI withdrawal is corroborated by the very highly significant reduction in chronic graft histopathology among those patients. To mitigate a possible impact on patient randomization resulting from not reaching the goal of CNI withdrawal in every patient so randomized, we censored from the analysis five patients in the CNI-minimized group due to early rejection (<6 months) because such patients were disqualified from withdrawal in the CNI-withdrawn group. We also included both intent-to-treat and on-treatment analyses as appropriate to aid in the interpretation of results.

This study suggests that the potential benefits to using maintenance immunotherapy with sirolimus (e.g., reduced risk of cancer) are best going to be appreciated if CNI levels are run very low or eliminated. Whether the improvement we observed in renal function after CNI withdrawal will impact long-term graft survival remains uncertain, but the reduced composite Banff injury score, less IFTA, and stable if not reduced total inflammation suggests that improved graft survival is possible. Future trials are warranted to determine the possible impact on long-term graft survival of CNI-free maintenance regimens.

## Supporting Information

S1 Protocol STAT trial protocol (*Prospective*, *Randomized Trial of Thymoglobulin* ^*®*^ *Induction Therapy for Renal Transplantation*: *Single vs*. *Alternate Day Administration*). (DOCX)

S1 Criteria Defining Criteria for Complications. (DOCX)

S1 Treatment Treatment of Acute Cellular Rejection after Renal Transplantation. (DOCX)

S1 Dataset De-identified essential data from the STAT trial. (XLS)

S1 CONSORT Checklist (DOC)
