# Supplementary material for: A Randomized 2x2 Factorial Clinical Trial of Renal Transplantation: Steroid-Free Maintenance Immunosuppression with Calcineurin Inhibitor Withdrawal after Six Months Associates with Improved Renal Function and Reduced Chronic Histopathology
Source: PLoS One. 2015 Oct 14;10(10):e0139247. doi: 10.1371/journal.pone.0139247 (PMC4605789; doi:10.1371/journal.pone.0139247)
Supplement: S1 Protocol — (DOCX) [file pone.0139247.s001.docx]

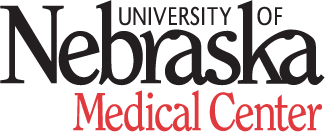


Institutional Review Board (IRB)

# IRB APPLICATION FOR BIOMEDICAL RESEARCH

SECTION I

Status:

___ New Submission

___ Five Year Resubmission (Current IRB # 286-03-FB)

**Title of Protocol:** Prospective, Randomized Trial of Thymoglobulin^®^ Induction Therapy for Renal Transplantation: Single vs. Alternate Day Administration

**Principal Investigator:** R. Brian Stevens, MD, PhD

**Secondary Investigator(s):**

Lucile E. Wrenshall, MD, PhD; Wendy Grant, MD; Jean Botha, MB, BCh; Alan Langnas, DO; Byers Shaw, Jr., MD;; Jennifer Larsen, MD; James Lane, MD; Troy J. Plumb, MD; David Mercer, MD; Michael Morris, MD, Vinaya Rao, MD; Clifford Miles, MD

**Participating Physicians/Health Care Personnel:**

Anna Kellogg, RN, BSN, MS; Sue Miller, RN, CCTC; Anna McGrain, MSN, APRN-BC; Sally Jane Guthmiller, MPAS, PA-C; Kristin Boothby, MS, APRN; Kecia Christiansen, MSN, APRN; Shannon Yannone, APRN; Kris Seipel, CCRC,

**Data and Administrative Personnel:**

Theodore Rigley, Kathleen Nielsen, Jill Skorupa, Thomas Veys, Anna Skorupa, Anthony Murante

Contact Person (PI or Coordinator): Anna Kellogg, RN, BSN, MS

Contact Person Phone and Pager: 559-3296; 888-0744

Contact Person e-mail: amkellogg@unmc.edu

PI Department: Surgery - Transplant

PI Phone: 9-2556

PI e-mail: rbstevens@unmc.edu

PI Address and campus ZIP: UMA 4525; zip: 3285

Funding Source: Check all that apply and supply names

___ Grant – Provide source:

**X**  Commercial – Provide company name: **Genzyme**

___ Other – Provide source:

___ None

Funding agency deadline for IRB approval: **NA**

Performance sites (see instructions): **UNMC/ The Nebraska Medical Center**

# IRB APPLICATION FOR BIOMEDICAL RESEARCH

SECTION I

**Research Characteristics Checklist**

**Special Populations** (chose all that apply)

__ Minors

___ Pregnant women

___ Fetuses

___ Prisoners (*must attach addendum for Research Involving Prisoners*)

___ Decisionally Impaired

**Special Categories**

___ Genetic Testing (*must attach addendum for Research Involving Genetic Testing*)

___ Gene Transfer (*must submit application to IBC*)

___ Xenotransplantation (*must submit application to IBC*)

**Drugs/Devices** (Choose all that apply)

___ No drugs are used for research purposes

X__ Marketed drugs (*must submit Marketed Drug Form to P&T Committee*)

___ Investigational drug (*must submit Investigational Drug Form to P&T Committee*)

IND # ____________

IND # ____________

___ Investigational Device

IDE # ____________

___ Significant Risk (see Investigator’s Brochure)

___ Non-Significant Risk

**Additional Review Requirements** (Choose all that apply. See individual committee submission requirements)

___ Departmental or other Peer Review Committee

___ Eppley Cancer Center Scientific Review Committee (SRC)

X__ Pharmacy & Therapeutics Committee

___ Institutional Biosafety Committee

___ Radioactive Drug Research Committee

**3. PRINCIPAL INVESTIGATOR'S ASSURANCE**

**The Principal Investigator understands and accepts the following obligations to protect the rights and welfare of research subjects in this study:**

I recognize that as the Principal Investigator it is my responsibility to ensure that this research and the actions of all project personnel involved in conducting the study will conform with the IRB approved protocol, IRB requirements/policies, and all applicable HHS/FDA regulations.

I recognize that it is my responsibility to ensure that valid informed consent/assent has been obtained from all research subjects or their legally authorized representatives. I will ensure that all project personnel involved in the process of consent/assent are trained properly and are fully aware of their responsibilities relative to the obtainment of informed consent/assent according to the IRB guidelines and applicable federal regulations.

I will inform the IRB of any unanticipated adverse event or injury in the time frame defined by IRB Guidelines and Policies.

I will not initiate any change in protocol without IRB approval except when it is necessary to reduce or eliminate a risk to the subject, in which case the IRB will be notified as soon as possible.

I will maintain all required research records on file and I recognize that the IRB is authorized to inspect these records.

I certify that there are adequate resources and facilities to carry out this research, including staff, funding, space, record keeping capability, and resources to address adverse events and possible research related injuries.

I will inform the IRB immediately of any significant negative change in the risk/benefit relationship of the research as originally presented in the protocol and approved by the IRB.

I understand that IRB approval is valid for a maximum period of five years with continuing review by the IRB required at least annually in order to maintain approval status.

I understand that I am responsible for appropriate research billing by enrolling patients using only the company center number assigned to this study.

I will inform the IRB immediately if I become aware of any violations of HHS regulations (45CFR46), FDA regulations (21CFR50,56) or IRB requirements for the protection of human subjects.

I understand that failure to comply with all applicable HHS/FDA regulations, IRB requirements/policies and the provisions of the protocol as approved by the IRB may result in suspension or termination of my research project.

Signature of Principal Investigator Position Date

**4. SECONDARY INVESTIGATOR CERTIFICATION**

Signatures of all secondary investigators must be obtained prior to IRB granting final approval and release of the protocol. In order to obtain the required certification of all investigators, multiple copies of this form may be submitted as necessary, or individual certifications of investigators can be transmitted to the IRB by letter.

Principal Investigator: R. Brian Stevens, MD, PhD

Title of Protocol: Prospective, Randomized Trial of Thymoglobulin Induction Therapy for Renal Transplantation: Single vs. Alternate Day Administration

Secondary Investigators:

| Lucille E. Wrenshall, MD, PhD | ___________________________ | ______________ |
| --- | --- | --- |
| Printed Name of Investigator | Signature | Date |
| Wendy Grant, MD_________ | ___________________________ | ______________ |
| Printed Name of Investigator | Signature | Date |
| Jean Botha, MB, BCh______ | ___________________________ | ______________ |
| Printed Name of Investigator | Signature | Date |
| Alan Langnas, DO_________ | ___________________________ | ______________ |
| Printed Name of Investigator | Signature | Date |
| Byers Shaw, Jr., MD________ | ___________________________ | ______________ |
| Printed Name of Investigator | Signature | Date |
| Gerald Groggel, MD________ | ___________________________ | ______________ |
| Printed Name of Investigator | Signature | Date |
| Jennifer Larson, MD_______ | ___________________________ | ______________ |
| Printed Name of Investigator | Signature | Date |
| James Lane, MD__________ | ___________________________ | ______________ |
| Printed Name of Investigator | Signature | Date |
|  |  |  |
|  |  |  |
| Troy J. Plumb, MD________ | ___________________________ | ______________ |
| Printed Name of Investigator | Signature | Date |
| David Mercer, MD________ | ___________________________ | ______________ |
| Printed Name of Investigator | Signature | Date |
| Michael Morris, MD_______ | ___________________________ | ______________ |
| Printed Name of Investigator | Signature | Date |
| Vinaya Rao, MD­­­­­­_________ | ___________________________ | ______________ |
| Printed Name of Investigator | Signature | Date |
| Clifford Miles, MD________ | ___________________________ | ______________ |
| Printed Name of Investigator | Signature | Date |

Signature certifies that the secondary investigator listed above has reviewed the protocol and will comply with all applicable HHS/FDA regulations and IRB requirements and policies for protection of human subjects.

**5. SCIENTIFIC/SCHOLARLY MERIT AND RESOURCE REVIEW CERTIFICATION**

*Research proposals must undergo substantive scientific and scholarly merit and resource review* ***prior to submission*** *of the application to the IRB. This IRB Application must provide evidence of this review. IRB applications submitted without this certification will be returned to the principal investigator without IRB review.*

*The chairperson, authorized delegate, or appointed review committee of the principal investigator’s school, department or division is responsible for review of the research proposal prior to submission.*

*The following attestation statement* ***must*** *be signed:*

My signature certifies that this protocol has been reviewed for scientific and scholarly merit and has been found to be acceptable. Additionally I certify that the principal investigator has the appropriate qualifications, experience, and resources to conduct this study.

________________________________

Signature of Reviewer

________________________________

Printed Name of Reviewer

________________________________

Position

________________________________

Date

**SECTION II**

**PROTOCOL ABSTRACT**

Title of Protocol: Prospective, Randomized Trial of Thymoglobulin^®^ Induction Therapy for Renal Transplantation: Single vs. Alternate Day Administration

Rabbit anti-thymocyte globulin (rATG, Thymoglobulin^®^) is a polyclonal anti-thymocyte preparation which effectively treats rejection of transplanted kidneys, and is used by many transplant physicians as “induction” therapy at the time of kidney transplantation. Although this preparation is usually administered in a series of divided doses over a course of several days following transplantation, evidence exists that concentrating the administration of a course of rATG at the time of transplantation may confer additional benefits to renal function and possibly increase resistance of the transplanted kidney to calcineurin nephrotoxicity. We hypothesize that a single dose of rATG administered over 24 hours at the time of transplantation will prevent rejection as adequately and safely as the conventional divided dose administration but result in better renal function and graft survival. Testing of this hypothesis will occur via assessment of distinct clinical and immunologic outcomes, outlined in this proposal, in adult recipients of kidney transplants.

The specific primary objectives of this research are to compare the safety and efficacy of single dose (6 mg/kg) rATG induction with the same total dose (6 mg/kg) administered every other day for four doses and to compare calcineurin inhibitor continuation versus calcineurin inhibitor withdrawal. The secondary objectives are to determine the immunological consequences of single dose (6 mg/kg) versus multiple dose administration of rATG; immunological consequences of calcineurin inhibitor continuation versus withdrawal; and to perform a cost analysis of single dose vs. multiple dose administration of rATG.

Eligible subjects will be receiving a kidney transplant for the first time or a subsequent re-transplant if certain criteria are met. Subjects must not require treatment with steroids for any other medical condition. Patients nineteen years of age and older are eligible. Subjects will be randomized into four treatment groups in a 1:1:1:1 ratio. All subjects will receive maintenance immunosuppression of tacrolimus (Prograf) and sirolimus until six months after transplant. At that time, subjects randomized to Groups 3 and 4 may begin sirolimus and mycophenolate mofetil if they have not experienced an acute rejection in the first six months post-transplant.

All subjects will receive the standard follow-up care for kidney transplant recipients at this center. This includes clinic visits and laboratory evaluations to monitor their kidney function and current health status. All subjects will have a kidney biopsy at 6 and 12 months post-transplant. Subjects who present with a condition, either medically or anatomically, that would present a greater risk to the subject, may at the discretion of the Principal Investigator forego a kidney biopsy. Clinical information about each subject will be entered into a computer database in order to evaluate the outcomes of the research.

**PURPOSE OF THE STUDY AND THE BACKGROUND (1-2).**

**1. Purpose of the Study**

**What are the specific scientific objectives (aims) of the research?**

Primary goals:

1. Compare the safety and efficacy of single-dose (6 mg/kg) rATG induction with the same total dose (6 mg/kg) administered every other day for four doses (divided-dose).
2. Compare standard renal transplantation outcome measures in patients converted approximately 6 months post-transplantation from maintenance immunosuppression with sirolimus and tacrolimus to sirolimus and low-dose mycophenolate mofetil.

Secondary goals:

1. Determine the immunological consequences of single-dose (6 mg/kg) versus divided-dose administration of rATG
2. Determine the immunological consequences of calcineurin-inhibitor (CNI) continuation versus CNI withdrawal.
3. Perform a cost analysis of single-dose vs. divided-dose administration of rATG.

**2. Background**

**Describe the background of the study. Include a critical evaluation of existing knowledge, and specifically identify the information gaps that the project is intended to fill.**

Rabbit anti-thymocyte globulin (rATG, Thymoglobulin^®^) is a polyclonal anti-thymocyte preparation which effectively treats rejection of transplanted kidneys, and is used by many transplant physicians as “induction” therapy at the time of kidney transplantation. Although this preparation is usually administered in a series of divided doses over a course of several days following transplantation, evidence exists that concentrating the administration of a course of rATG at the time of transplantation may confer additional benefits to renal function and possibly increase resistance of the transplanted kidney to calcineurin nephrotoxicity. We hypothesize that a single dose of rATG administered over 24 hours at the time of transplantation will prevent rejection as adequately and safely as the conventional divided dose administration but result in better renal function and graft survival. Testing of this hypothesis will occur via assessment of distinct clinical and immunologic outcomes, outlined in this proposal, in adult recipients of kidney transplants.

The primary mechanism of action of rATG in vivo, determined by primate studies, is likely cell death via apoptosis. At high doses, cell death occurs in both the bloodstream and in lymph nodes, but not in the thymus. Based on their primate data, Preville, et al,^1^ surmised that lymphocyte depletion in tissues may correlate with peak levels of Thymoglobulin rather than the total dose given. Cell surface markers are down-modulated, in a dose-dependent fashion, on lymphocytes that have escaped deletion. In humans, Thymoglobulin^®^ induces short-term depletion of peripheral lymphocytes, and a long-term decrease in the CD4:CD8 ratio of T-cells in the periphery. Studies of immune cell reconstitution following chemotherapy or treatment of AIDS have revealed that restoration of the CD4+ T-cell compartment comes largely from the thymus. Restoration of the CD8+ compartment comes mainly from proliferation of cells present in the peripheral immune system, which likely explains the changes in CD4:CD8 ratio that are seen following Thymoglobulin. In addition to the changes in the CD4:CD8 ratios, patients treated with Thymoglobulin also exhibit a long-term increase in the proportion of CD8+ CD57+ T-cells. These T-cells represent a replicatively senescent population prone to activation-induced cell death, and are associated with improved graft survival in recipients of kidney transplants.

Although most centers administer rATG over several days, a few centers have reported their experience with a single bolus of anti-thymocyte globulin (ATG). Three centers used ATG in a single bolus of 9 mg/kg without untoward side effects. In these reports, a single bolus of ATG was compared to no induction therapy, and therefore no information can be gleaned as to potential differences between single-dose and divided-dose administration. Agha, et al,^2^ reported results in which a single dose of Thymoglobulin at 3 mg/kg was followed by 2 doses at 1.5 mg/kg. He compared this regimen retrospectively to the standard regimen of 1.5 mg/kg for 7-10 days. Interestingly, a more prolonged lymphocyte depletion was observed with the “short course” regimen containing the higher initial dose. Swanson, et al,^3^ reported the use of very large dose Thymoglobulin^®^ administered in a regimen of 2.5 mg/kg/day for a total dose of 20 mg/kg. Absolute lymphocyte counts at 6 months were similar to those observed by Agha, et al for patients receiving the “short course” induction therapy. Although the maintenance immunosuppression regimens were quite different (rapamycin versus cyclosporine, azathioprine, and prednisone), a comparison of these studies suggests that large total doses of Thymoglobulin, given in several smaller doses, may be of no greater efficacy than smaller total doses given as larger individual boluses. These findings may relate to the development of anti-rabbit antibodies. Preville, et al,^1^ noted that in primates Thymoglobulin antibodies were totally neutralized by anti-Thymoglobulin antibodies at 11 days post-initiation of treatment. In humans, Regan, et al,^4^ reported that 70% of patients developed anti-Thymoglobulin antibodies by 3 weeks post-initiation of multi-dose therapy. Since repeated administration of an antigen is the best way to initiate the formation of antibodies, a large single dose of Thymoglobulin may result in decreased sensitization and improved efficacy.

Given the long-term effects of rATG, administration of this agent may impact a patient’s requirements for maintenance immunosuppression. Therefore, if a single-dose of rATG is more efficacious than multiple, low doses of rATG, patients receiving this single dose may require less maintenance immunosuppression. To test this hypothesis, patients receiving single-dose or divided-dose rATG will also be randomized to either continue receiving sirolimus and tacrolimus maintenance immunosuppression or be converted after approximately 6 months to sirolimus and low-dose mycophenolate mofetil. Although the combination of tacrolimus and sirolimus has rendered excellent prophylaxis against rejection at our institution, this combination is not tolerated by all patients, especially with regard to side effects such as leukopenia, anemia, and hyperlipidemia.

In order to assess maintenance immunosuppression without the toxicities associated with calcineurin inhibitors on renal allograft function, monotherapy with sirolimus was initially considered for subjects randomized to two of the treatment groups. Subjects who were rejection-free at six months post-transplant would be eligible to change to this regimen. However, in one small study reported by Rao et al^5^, 29 primary kidney transplant patients received induction therapy with Campath-1H followed by monotherapy with sirolimus. In this group, 8 patients (28%) had rejection. Compared to Thymoglobulin, Campath-1H is a more potent induction agent, and thus our protocol may have a rejection rate in excess of 28%. If this were to be the expected rate of rejection, it would be unacceptable.

This investigator has also had numerous discussions with other members of the kidney transplant community who expressed concern that monotherapy with sirolimus may not provide enough protection against rejection. Instead, dual-therapy with sirolimus and low-dose mycophenolate mofetil would achieve the same goal of our original proposal, namely, elimination of chronic use of calcineurin inhibitors while still providing adequate protection against rejection. Finally, patients receiving this dual-therapy may be less susceptible to certain viral infections that are particularly problematic in renal transplant patients (especially CMV and BK virus).

**CHARACTERISTICS OF THE SUBJECT POPULATION (3-9)**

**3. Accrual**

**a. What is the maximum number of subjects that will be consented at UNMC, The Nebraska Medical Center or other sites approved by the UNMC IRB (that is, sites listed in Section I of this application)?** 180

**b. What is the statistical or other justification for the maximum accrual requested?**

A detailed analysis of the statistical power and sample size requirements of this study was prepared by Julie Stoner in July 2005, and is attached to this 5-year re-write in fulfillment of the requirements of this section. This statistical power and sample size analysis concludes that the study goals can be met with a total enrollment of 200 subjects, which is sufficient to compensate for a 10% drop-out rate. Our experience with this study to date appears to substantiate this enrollment size as being appropriate to meet study goals.

**c. For multicenter protocols, what is the total number of subjects to be enrolled at all sites?** NA – this is a single-center protocol.

**4. Gender of the Subjects**

**a. Are there any enrollment restrictions based on gender?**

No, both male and female subjects are eligible for enrollment.

**b. Are there any enrollment restrictions based on child-bearing potential?**

All sexually active kidney recipients of childbearing potential are strongly encouraged to use two reliable, medically acceptable forms of birth control prior to and following kidney transplantation while actively on immunosuppressant drugs. The side effects of these drugs on developing fetuses make this necessary. Female study participants who are not of reproductive potential (have reached menopause or undergone hysterectomy, oophorectomy, or tubal ligation) or whose male partner has undergone successful vasectomy with resulting azoospermia or has azoospermia for any other reason, are eligible without requiring the use of contraception. Male study volunteers, who have undergone successful vasectomy with resulting azoospermia or have azoospermia for any other reason, are eligible without requiring the use of contraception**.**

**c. Are there any specific contraception requirements?**

Two reliable, medically acceptable forms of birth control should be used prior to and following kidney transplantation while an individual of childbearing potential is actively on immunosuppressant drugs.

**d. Are pregnant or breast feeding women excluded from participation?**

As is standard of care for transplantation recipients, pregnant women may not participate in this study due to the potential teratogenic effects of immunosuppressant on developing fetuses.

**5. Age Range of Subjects**

**a. What is the age range of the adult subjects?**

Subjects will be between 19 and 65 years of age.

**b. What is the rationale for selecting this age range?**

Adults greater than 65 years of age are at an increased risk for the following complications surrounding transplantation and immunosuppressive therapy:

- Death due to cardiovascular disease
- Prolonged surgical recovery
- Infection and malignancy related to immunosuppression
- Slowed metabolism of immunosuppressive drugs
- Decline in cognitive function which may lead to inability to comply with post-transplant care.

Specific to this study, patients greater than 65 years of age may not be able to tolerate a single dose of rATG due to the propensity for decreased cardiac function.

The lower age limit was selected because in Nebraska the age of majority is 19 years old.

**c. Will children and/or adolescents (18 years of age or younger) be included in this research?**

No children and/or adolescents will be included in this research. The medications utilized in this study have not been well studied in children less than 18 years of age, resulting in a concern about the potential for a negative impact on development or unforeseen side effects.

**6. Race and Ethnicity**

**Are there any subject enrollment restrictions based upon race or ethnic origin?**  There are no enrollment restrictions based upon race or ethnic origin.

**7. Vulnerable Subjects**

**Will any of the following populations be specifically recruited for enrollment in this research?**

**a. Prisoners**

**b. Pregnant women or fetuses**

**c. Cognitively or decisionally impaired**

**d. Economically or socially disadvantaged**

**e. Terminally ill**

No vulnerable subjects will be enrolled in this study.

**8. Inclusion Criteria**

**What are the specific inclusion criteria?**

- 1. Primary renal transplant recipient
  2. Recipient of a re-transplant, if:
- previous graft(s) lost due to non–immunological factors, ***or***
- previous graft(s) lost due to immunological factors, but while maintained on the less-effective immunosuppression agents of the previous era, i.e., cyclosporine, steroids, and/or azathioprine
  1. Cadaveric donors
  2. Living renal donor
  3. Recipient of renal transplant with planned pancreas transplant at a later date
  4. Zero-mismatch human leukocyte antigen donors

**9.** **Exclusion Criteria**

**What are the specific exclusion criteria?**

1. Recipient age <19 years or >65 years
2. Previous history of CMV disease
3. Hepatitis B and C recipients
4. Primary disease states that require steroids for immunosuppression
5. Re-transplant with immunological cause of renal or pancreas loss
6. Non-heart beating donors
7. Recipient of pediatric en bloc kidneys
8. Recipient with a PRA >75%
9. Patients who have received 3 or more prior transplants, excluding pancreas
10. Patients who are past recipients of other solid organ transplants
11. Previous history of BK virus
12. Previous treatment with rATG
13. Allergy to rabbits
14. Simultaneous Kidney/Pancreas transplantation

**METHODS AND PROCEDURES (10-12)**

**10. Methods and Procedures Applied to Human Subjects**

a. Briefly describe the study design (e.g. randomized, double blind, cross-over, phase III).

This is a prospective, randomized, non-blinded 2 x 2 factorial comparison of clinical outcomes in 180 renal transplant recipients receiving either a single dose of rATG at 6 mg/kg or 4 doses given every other day at 1.5 mg/kg/dose. Immediately after renal transplantation, each of these groups will undergo our clinically-standard early steroid withdrawal; approximately 6 months later, half the subjects in each group will be switched to maintenance immunosuppression using Sirolimus/MMF.

**b. Are there any tests performed prior to signing of the consent form in order to determine eligibility for this research protocol?** No.

**c. Describe sequentially all procedures, interventions and evaluations to be applied to subjects, and identify any that are experimental or performed exclusively for research purposes.**

Participants must sign a written consent form prior to initiation of any study procedure.

At the time of transplantation, the participant will be randomized to one of 4 groups: (1) single-dose rATG at 6 mg/kg plus rapamycin and tacrolimus maintenance immunosuppression, (2) divided-dose (4 doses) rATG given every other day at 1.5 mg/kg/dose plus rapamycin and tacrolimus maintenance, (3) single-dose rATG plus rapamycin and tacrolimus maintenance with conversion after 6 months to rapamycin and mycophenolate mofetil maintenance, and (4) divided-dose rATG plus rapamycin and tacrolimus maintenance with conversion after 6 months to rapamycin and mycophenolate mofetil maintenance. Given the long-term effects of rATG, single-dose versus divided-dose administration may have a differential impact on the patient’s requirements for maintenance immunosuppression (see section II.2). For this reason, we are randomizing patients to the 4 treatment groups delineated above.

Participants will receive all standard post-transplant clinical care and follow-up. This includes receiving both tacrolimus and rapamycin for the first 6 months post-transplantation. At the month 6 time point, the investigator will determine if it is safe for those subjects in groups 3 and 4 to transition to rapamycin and mycophenolate mofetil for maintenance immunotherapy. Eligible subjects will have had no rejection episodes in the first six months and will be able to tolerate a dose of rapamycin that achieves a trough level of no less than 8 ng/ml.

Participants will have renal function determined by estimated glomerular filtration rate (GFR) calculated according to the formula currently recommended by the National Kidney Foundation^6,7,8^. These GFR assessments will be made daily during the first one to three weeks post-transplantation, becoming monthly thereafter. 24-hour urine creatinine clearance at 1, 3, 6, 12, and 24 months may also be used to assess graft function. The creatinine clearance test is standard practice. There are a number of factors which can affect the accuracy of the 24 hour urine collection, such as misinterpretation of the instructions provided to patients regarding method of collection; temperatures at which the samples are maintained; and a voiding that is not collected, thus yielding an altered result. The calculated GFR will provide an accepted, objective measure for comparison among the groups.

Participants will undergo assessment of several immunologic parameters to assess potential mechanisms of action of each of these dosing regimens. Each of the parameters will be tested from peripheral blood samples obtained during routine clinical visits.

To measure the long-term effect of rATG, CD3 and CD4/CD8 counts with ratio will be done at baseline, two weeks post transplant, 1, 3, 6, 12, and 18 months, and annually to year 5. These tests are performed for research purposes.

Participants may be asked to have this additional blood drawn in order to test for certain immunological parameters. This will depend on whether or not the patient is able to return to the transplant center at the required intervals. The investigator, in consultation with the patient and the study coordinator, will determine if this is possible.

The following blood tests will be performed to determine if T-cells are being re-educated in the thymus following rATG administration:

- Transglutaminase expression will be measured 1 hr post-initiation of first dose of rATG.

● Serum levels of ε (γ-glutamyl)-lysine diisopeptide will be measured every other day for 7 days post-transplantation then weekly until pre-transplant levels are reached.

● TREC output will be measured weekly, until levels return to baseline, beginning 2 days post-completion of rATG therapy.

● CD3 counts will be measured daily until total counts are > 100.

● Gene array analyses for assessment of functional signal transduction pathways and analysis of cell surface phenotypes of peripheral blood leukocytes will be performed at 1, 3, 6, and 12 months post-transplantation.

All participants will follow the standard post-transplant schedule for routine laboratory testing.

For safety purposes, subjects will have surveillance biopsies at 6, 12, and 24 months. If future funding becomes available, a renal biopsy at year 5 may be added. (If this becomes feasible, the protocol will be amended and an addendum consent form will be prepared for the subjects.) Subjects who present with a condition, either medically or anatomically, that would present a greater risk to the subject, may at the discretion of the Principal Investigator forego a kidney biopsy. Banff scoring will be utilized to categorize rejection and transplant nephropathy. It is standard practice at this center to request surveillance biopsies for renal transplant recipients who are not receiving steroids as part of their maintenance immunosuppression.

Participants will return for study visits at 1, 3, 6, 12, and 24 months and as needed in accordance with standard schedule for all transplant patients. Long-term data regarding graft and patient survival, incidence of allograft rejection, serious adverse events, CMV disease, opportunistic infections, PTLD, hyperlipidemia, wound healing problems, lymphoceles and immunosuppression regimen will be collected at years 3, 4, and 5 from a review of the subject’s medical record.

Adverse Events:

All adverse events will be monitored and recorded. These will include both events related to the safety profile of the study drugs (CMV disease, Post-Transplant Lymphoproliferative Disorder [PTLD], opportunistic infections, elevated lipids, etc.) and expected renal transplant complications (wounds, lymphoceles, diabetes, weight gain, etc.). All unexpected adverse events and any deaths will be reported to the IRB per UNMC policies.

Endpoints:

Primary endpoints:

1. Cumulative calcineurin nephrotoxicity/ transplant nephropathy per clinically-indicated and protocol surveillance biopsies (Banff grading criteria)
2. Renal function by either measured 24-hour urine creatinine clearance at 1, 3, 6, 12 and 24 months or calculated GFR (Glomerular Filtration Rate) by using the abbreviated MDRD (aMDRD) formula and patient serum creatinine and demographic data. This may be calculated from routine blood draws, allowing multiple data points to be collected within each month.

## Secondary endpoints:

1. Safety profile

1. CMV disease

2. Opportunistic infections,

3. PTLD

4. Hyperlipidemia

5. Wound healing problems

6. Lymphoceles

1. Requirement for additional immunosuppression (such as corticosteroids, antimetabolites or other immunosuppressive agents)
2. Acute rejection per kidney biopsy (Banff grading criteria)
3. ATN (acute tubular necrosis) rate (requirement for dialysis within 7 days post-transplant)
4. Graft survival
5. Patient survival
6. Immunological parameters
7. New onset BK virus per kidney biopsy
8. **Describe the follow-up of subjects and identify any procedures that are performed exclusively for research purposes, or performed more frequently than would normally be clinically indicated (e.g. additional or more frequent radiologic evaluations).**

All participants will follow the standard post-transplant schedule for routine laboratory testing.

For safety purposes, subjects will have surveillance biopsies at 6, 12, and 24 months. The renal biopsy at 24 months following transplant is performed exclusively for research purposes. Subjects who present with a condition, either medically or anatomically, that would present a greater risk to the subject, may at the discretion of the Principal Investigator forego a kidney biopsy. It is standard practice at this center to request surveillance biopsies for renal transplant recipients who are not receiving steroids as part of their maintenance immunosuppression.

Participants will return for study visits at 1, 3, 6, 12, and 24 months and as needed in accordance with standard schedule for all transplant patients. Long-term data regarding graft and patient survival, incidence of allograft rejection, serious adverse events, CMV disease, opportunistic infections, PTLD, hyperlipidemia, wound healing problems, lymphoceles and immunosuppression regimen will be collected at years 3, 4, and 5 from a review of the subject’s medical record.

To measure the long-term effect of rATG, CD3 and CD4/CD8 counts with ratio will be done at baseline, two weeks post transplant, 1, 3, 6, 12, and 18 months, and annually to year 5. These tests are performed for research purposes.

Participants may be asked to have this additional blood drawn in order to test for certain immunological parameters. This will depend on whether or not the patient is able to return to the transplant center at the required intervals. The investigator, in consultation with the patient and the study coordinator, will determine if this is possible.

The following blood tests will be performed to determine if T-cells are being re-educated in the thymus following rATG administration:

- Transglutaminase expression will be measured 1 hr post-initiation of first dose of rATG.

● Serum levels of ε (γ-glutamyl)-lysine diisopeptide will be measured every other day for 7 days post-transplantation then weekly until pre-transplant levels are reached.

● TREC output will be measured weekly, until levels return to baseline, beginning 2 days post-completion of rATG therapy.

● CD3 counts will be measured daily until total counts are > 100.

● Gene array analyses for assessment of functional signal transduction pathways and analysis of cell surface phenotypes of peripheral blood leukocytes will be performed at 1, 3, 6, and 12 months post-transplantation.

**e. Describe briefly the statistical methods used to analyze the data.**

Binomial and numerical outcomes are described using proportions and means, respectively, and are compared using chi-square test or Fisher’s exact test when appropriate and t test, respectively. Graft survival and acute rejection rates are determined using Kaplan-Meier estimates and are compared using log-rank tests. The effects of covariates on the hazard risk of graft survival and acute rejection are evaluated using a Cox proportional hazard model.

A mixed-model for repeated measures is used to compare calculated GFR values to examine the effect of single versus divided-dose rATG on kidney function. A linear mixed-model of repeated measures is used to examine the relationship between the amount of rATG infused at the time of reperfusion and calculated GFR during the follow-up period.

**f. Does this research involve genetic testing?**

Genetic testing will not be involved in this research protocol.

**g. Does this research involve banking of information or material (blood, tissue, etc) for future use or for purposes that are not integral to the current research?**

This research involves the collection and storage of data for comparison between enrolled study subjects. This information to be collected and analyzed is data that are currently collected and stored as part of the standard clinical care that a transplant recipient receives. Gathering of this information will continue as long as the graft is functioning or until the subject passes away and may be used in future publications. This project does not involve the banking of blood, tissues, etc.

**11. Drugs and Devices**

**a. Does this study involve investigational drugs or devices (test articles)?** This study does not involve investigational drugs or devices.

**b. Does this study involve FDA approved drugs or devices used for off-label purposes?**

Yes, the FDA-approved drug Thymoglobulin will be used off-label.

**1) What is the off-label use?** Off-label use of Thymoglobulin is for immunosuppression induction.

**2) Will the results of this study be used by the manufacturer to support (a) a new indication for the drug, (b) a significant change in the labeling for the drug, or (c) a significant change in the advertising for the drug?** Based on study findings to date, the manufacturer has indicated interest in obtaining a new label indication for Thymoglobulin use as an induction agent in transplantation. Such an additional indication will require a more extensive multi-center trial for which the current study can be viewed as a pilot study.

**3) Does the study involve a route of administration or dosage level, use in a subject population, or other factor that significantly increases the risks (or decreases the acceptability of the risks) associated with the use of the drug?**

No, our study results to date indicate that the risks associated with the administration of the single rATG dose are statistically identical to the risks associated with the administration of the conventional divided-dose.

**c. Provide an FDA Use-in-Pregnancy category (A, B, C, D or X) for each drug used as a part of this research.**

The FDA Use-in-Pregnancy category for Thymoglobulin is C.

**12. Data Storage and Confidentiality**

**a. Where will the research data be stored during the study and how will it be secured?**

Research data will be collected and stored in the Department of Surgery in an office that is locked during non-business hours on computers that are password-protected. Information obtained through this research protocol will be available only to qualified investigators, the study team, representatives of the IRB, and to persons involved in the care of these patients. No patient data will be released to unqualified individuals and no data with subject identifiers will be released in reports or published.

**b. Will research data be disclosed to anyone at UNMC and The Nebraska Medical Center who is not listed in section I of the application?**

Yes, the research data may be disclosed to members of the IRB upon request.

**c. Will research data be disclosed to any investigators outside of UNMC and The Nebraska Medical Center?**

Yes, research data without patient identifiers will be disclosed to John Sandoz of Sigma Analytics, who will be performing biostatistical analyses for us under contract.

**d. Will research data be disclosed to any commercial sponsor, contract research organization (CRO) or Data and Safety Monitoring Board (DSMB)?**

No.

**e. Will research data be disclosed to any other external organization or entity (e.g., cooperative groups, NIH, FDA, registries)?**

The research data may be disclosed to the FDA; however disclosure of this data will not include patient specific identifiers.

**f. Will research data be shared with third party payers?** No.

**g. For what duration of time will research data be subject to disclosure to the persons or groups identified above?**

The research data will be subject to disclosure for the duration of the research and until data analysis is complete.

**RISK/BENEFIT ASSESSMENT (13-19)**

**13. Potential Risks**

**What are the potential risks associated with each intervention? If data are available, estimate the probability that a given harm may occur and its potential reversibility.**

Thymoglobulin: The study drug, Thymoglobulin, works by destroying one type of the body’s white blood cells (lymphocytes) that fight against the transplanted organ as well as infection. Thymoglobulin can also cause a decrease in the number of platelets. This may increase the risk of bleeding after surgery. Investigators will monitor the number of white blood cells and platelets every day while the subject is receiving the study drug, and the Thymoglobulin dose may be adjusted if blood counts fall. The investigator may stop Thymoglobulin if they fall too low, and Thymoglobulin may be restarted once these levels come back up.

Rarely, the symptoms of serum sickness (rash, muscle aches, fever and joint pain) can occur approximately 2 weeks after therapy with Thymoglobulin. These symptoms can be treated with administration of steroids, medications that the patient will receive as part of the transplant therapy during his/her initial post transplant hospital stay.

In rare instances, the patient may experience an allergic reaction to the study medication. This occurs because he/she is receiving a foreign protein (a product produced in a rabbit). The allergic reaction may cause a difficulty in breathing, low blood pressure, and shock.

While the patient is receiving the Thymoglobulin, he/she will be monitored closely with frequent measurements of pulse, breathing, and blood pressure. Severe lung (respiratory) reactions or allergic reactions are extremely uncommon but can be potentially life threatening.

Very common adverse events occurring in greater than 10% of patients who participated in a clinical study include: decreased white blood cells, decreased platelets (needed for normal blood clotting), headache, joint pain, and back pain. Nausea, vomiting, fever, chills, and increased heart rate have also occurred which may or may not be a “first dose effect” from the initial Thymoglobulin administration. The patient will be given pre-medications prior to the administration of each dose of Thymoglobulin to minimize these symptoms.

Common adverse events occurring between 1 and 10% of patients who participated in a clinical study include: diarrhea, chest pain, tremors, shortness of breath, decreased blood pressure, decreased or lack of appetite, dizziness, itching, sweating, and hives. Rash and/or muscle aches have also occurred which may or may not be symptoms of serum sickness.

The use of Thymoglobulin may increase the risk for certain types of infections (bacterial, viral, and fungal) after transplantation. These risks may be further increased by the administration of Thymoglobulin. Types of infections reported following the use of Thymoglobulin include viral infections such as Epstein-Barr virus (EBV) infection and cytomegalovirus (CMV) infection, fungal infections such as aspergillosis, and bacterial infections. Other infections have been reported systemically in the blood or tissues.

The risk of developing certain kinds of tumors is increased after transplantation associated with the use of immunosuppression, such as is being employed in both arms of this study. Lymphomas secondary to an EBV infection are a common malignancy reported following transplant and intense immunosuppression. Other malignancies have been reported on the surface of the skin and lungs, and less commonly other organs.

Patients may experience some pain and bruising at the site where the Thymoglobulin is being injected (infusion site in vein or central line).

Side effects (adverse events) experienced by patients who have recently received Thymoglobulin commercially (outside of formal clinical trials) are the following: multi-organ failure, respiratory failure, heart attack, complications of transplant surgery, complications with normal blood clotting, allergic reaction, accumulation of excess fluid in the lungs, pneumonia (both bacterial and viral), dehydration, decreased oxygen levels in blood and tissues, complications of kidney transplant such as renal tubular necrosis, and elevations in various liver and kidney function tests. As Thymoglobulin is approved and available commercially in many countries including the US and Canada, the exact frequency of these reported events cannot be determined.

Rapamune^®^: The most common side effects of Rapamune include a temporary increase in the blood levels of liver enzymes (showing an unwanted effect on the liver), and in cholesterol and triglycerides (fat in the blood). Temporary decreases in platelets (cells that help blood to clot), white blood cells (cells that fight infection), and in blood levels of phosphorus and magnesium have also occurred.

Some patients have also developed nose-bleeds, headache, indigestion, diarrhea, irritation of the mouth, anemia, night sweats, decreased male hormone, joint pain, infections, elevation of blood pressure, acne, rash, abnormal collection of lymph fluid around the transplanted kidney (lymphocele), fever, and bleeding abnormalities.

All patients will receive the standard medications needed to prevent the common infections after kidney transplant. Risk of monotherapy with Rapamycin could increase the risk of rejection in the transplanted organ.

Prograf^®^: The most common adverse effects of Prograf reported in kidney transplantation are tremor, headache, diarrhea, constipation, abdominal pain, high blood pressure, decreased kidney function, infection and insomnia. Other side effects that may occur with Prograf include numbness or tingling usually of the hands and feet, nausea and vomiting, lowered blood magnesium, decrease in the red blood cells, urinary tract infection, muscle weakness, shortness of breath, pain, fever, swelling, and skin rash or itching. Some patients may also experience elevated blood sugar levels and some of these patients will require insulin to control their blood sugar levels.

CellCept^®^: The principal adverse reactions associated with the administration of CellCept include diarrhea, leukopenia, sepsis, and vomiting. There is also evidence of a higher frequency of certain types of opportunistic infections.

Anti-rejection (immunosuppressant) medication: Other possible side effects associated with anti-rejection medicines are shortness of breath, itching or rash on the skin, fever or back pain.

The risk of developing certain types of tumors (cancer) after transplantation may be increased with the use of any of the anti-rejection medicines that are being used for all patients participating in this study.

Kidney biopsies: Kidney biopsies can be painful where the needle enters the body, and a bruise can form. Bleeding can occur where the needle is inserted into the kidney. Blood can appear in the urine.

Drawing blood: The usual risks of drawing blood include pain, possibility of infection, perforation of the vein, bruising, and/or discoloration or bleeding from the place where the needle was inserted. There is also a slight possibility of the formation of a blood clot or that you might feel faint. There is no additional risk when an extra tube of blood is drawn at the time of a routine blood draw.

Randomization: When patients are randomized to different groups, there is the risk that patients in one group will not do as well as patients in the other group. In this study, that could mean that one group may have a higher risk of developing rejection of the transplanted kidney than another group.

Pregnancy: Adequate, well-controlled human studies are lacking. Therefore, there may be risk to a fetus if these drugs are administered during pregnancy (FDA Use-in-Pregnancy Category C).

Unknown risks: The drugs used in this study may also involve risks that are not yet known.

**14. Risk Classification**

**What is the overall risk classification of the research: minimal risk, greater than minimal risk but less than significant risk, or significant risk?**

The risk classification is significant due to the nature of kidney transplantation and the continuing requirement for immunosuppression to preserve the graft.

**15. Data and Safety Monitoring**

**a. How will subject safety be assessed on an ongoing basis throughout the study?**

All subjects will be scheduled for follow up visits as outlined previously, which is standard practice in renal transplant. Investigators and participating healthcare providers will be present at the follow-up visits. Additionally, post-transplant coordinators will be following subjects, as is standard practice, through phone calls and other communications. Laboratory tests and biopsies performed at the various visits throughout the study will also allow for assessment of subject safety.

**b. What specific procedures will be utilized to prevent or minimize potential risks or discomforts associated with study interventions and procedures?** Thymoglobulin^®^; Prograf^®^; Rapamune^®^: CellCept^®^. Participants will be monitored closely for infections, malignancies, and any other adverse reactions.

Kidney biopsies: Precautions typically taken to minimize the risks of a biopsy include obtaining the following tests prior to the biopsy: a complete blood count, coagulation factors, renal ultrasound, and urinalysis. In addition, the biopsy is performed under the guidance of ultrasound.

To prevent bleeding at the site after the biopsy, patients are required to remain in bed for 6 hours and to lay for 4 hours following the procedure. A 5 lb. sandbag is placed over the site. Patients are closely observed and vital signs are checked regularly during this time.

Drawing blood: All usual precautions will be taken.

Randomization: All groups of subjects will receive standard maintenance therapy of tacrolimus and rapamycin in the early post-transplant period (i.e. to month 6). Only those randomized to groups 3 and 4 who have not experienced rejection and who are able to maintain rapamycin trough levels of 8 ng/ml or greater, will be transitioned to rapamycin and mycophenolate mofetil.

Pregnancy: All sexually active participants are required to practice two methods of medically acceptable birth control as long as they are on study medication and for 3 months following the discontinuation of study medication. For female subjects, pregnancy tests will be done prior to start of study medication, and if a pregnancy occurs, the participant will be withdrawn from the study.

Unknown risks: Participants are instructed to report any and all symptoms or reactions to the investigator or study staff. If side effects are unacceptable, the participant may be withdrawn from the study.

Other precautions: Procedures utilized to minimize risk include provision to the subject of contact phone numbers for coordinators and study personnel, administration of tests or interventions by qualified personnel, and the use of procedures and medications to reduce known side effects.

**c. What are the specific subject withdrawal criteria?**

Criteria for subject withdrawal from this study include:

- Withdrawal of consent by the subject at any time during the course of the study
- Second transplant after initial kidney transplant (i.e. pancreas and liver)
- Graft loss
- Subject is lost to follow-up
- Pregnancy
- Patient experiences an unmanageable adverse event
- Death of subject
- Study is terminated

**d. What are the specific stopping rules for the research?**

Interim Analysis and Criteria For Halting The Study

During the course of the trial we have monitored for adverse events potentially related to the immunosuppressive agents used in each of the four arms of the study. Interim analyses performed after enrollment of 60 and 140 patients have shown not only generally low and acceptable rates of study-drug related adverse events, but that specifically the rates of adverse events are statistically identical between the study arms.

The originally approved stopping rules for this study have become effectively obsolete, as the study has matured beyond the point where there is any possibility that additional adverse events could exceed the number prescribed as tolerable under the stopping rules algorithm.

While we consider that the safety of the immunosuppressive agents being used in this trial has been well demonstrated, we will continue the policy of stopping enrollment in the study, pending a complete investigation by the Principal, Co-principal, and Secondary investigators, if either of the following should occur:

1. Death of one subject due to an adverse drug reaction
2. A probability of life life-threatening, adverse drug reactions, including pulmonary edema requiring intubation, myocardial infarction, or hypotension requiring significant drug treatment.

Conditions that may warrant termination of the study include, but are not limited to:

- The discovery of an unexpected, serious, or unacceptable risk to patients enrolled in the study.
- The decision on the part of the Investigator to suspend or discontinue testing, evaluation, or development of the clinical program.
- Insufficient adherence to protocol requirements.

Study termination and follow-up will be performed in compliance with the conditions set forth in 21 Code of Federal Regulations (CFR) 312.

**e. Is there a DSMB for this research study?** No.

-If **no**, describe the process of ongoing data analysis:

**1) What is the frequency of data analysis?**

A formal interim analysis of the accumulated study data was conducted after accrual of 60 subjects and again at 140 subjects. The findings of the second 140 subject interim analysis were peer-reviewed and published in the journal *Transplantation*. Outcome data are continually being collected and audited as subjects progress through the initial subsequent phases of the study. An additional formal analysis of the induction phase will be performed at the accrual of the final study subjects. The optimal timing of a formal analysis of the outcome of the calcineurin inhibitor withdrawal phase will be defined as further experience of calcineurin inhibitor withdrawal accumulates.

**2) Who will perform the analysis?**

The initial interim analysis of the first 60 subjects was performed by Jeff Sun of the UNMC College of Public Health Department of Biostatistics. The second interim analysis of the 140 subjects was performed jointly by staff of the Kidney/Pancreas Transplant Program (Kathleen Nielsen, Megan Henning, and Theodore Rigley) and John Sandoz of Sigma Analytics. Ongoing analyses of further study data will be performed by this same group.

**16. Potential Benefits to the Subject**

**What are the potential benefits to the subject associated with the research?**

If the participant is randomized to the group which receives the single-dose of rATG, and this dose proves to promote tolerance of a transplanted kidney, then the participant may experience this benefit.

If the participant is randomized for conversion to sirolimus and low-dose mycophenolate mofetil maintenance immunosuppression beginning at 6 months, and this proves to be effective in preventing rejection of the transplanted kidney, they may benefit from decreased cost of medicines, and lower exposure to potentially serious side-effects of tacrolimus.

Having a kidney biopsy at 24 months will provide the subject’s physicians with additional information about the transplanted kidney. If kidney rejection is detected, then this biopsy may allow the physicians to begin anti-rejection treatment earlier. In addition, if biopsy results show moderate to severe chronic allograft nephropathy, the physicians would be able to adjust the immunosuppression therapy to minimize progression of the nephropathy.

The participant may experience no direct benefit.

**17. Potential Benefits to Society**

**What are the potential benefits to society that may result from this research?**

This study may help future patients who undergo kidney transplantation.

**18.** **Alternatives to Participation**

**a. What are the alternatives available to the subject in the *non-research* context which may be of reasonable benefit to the subject?**

**Standard clinical practice at UNMC for kidney transplant recipients is rATG given in four doses every other day starting on the day of transplant. Patients who do not wish to participate in this protocol would still receive the standard rATG dosing.** Standard maintenance anti-rejection treatment following kidney transplant is tacrolimus or cyclosporine with or without one or more of the following: corticosteroids (prednisone, prednisolone), mycophenolate mofetil and/or sirolimus.

**b. Would any of the interventions in the study be available to the subject if they did not elect to participate in the study?**

No, rATG induction given as a single-dose is available only in the context of this research study. Subjects who chose not to participate would receive standard of care immunosuppression induction, which currently is four doses of rATG given every other day beginning the day of transplant.

**19*.*** **Risk-Benefit Relationship**

**a. What is the relative risk-benefit relationship of the research?**

The primary benefit to patients receiving the single dose of rATG may be an increase in tolerance of the graft. If this occurs, these patients may require reduced levels of maintenance immunosuppression. This would translate into less exposure to potentially toxic side effects and reduced cost.

Further, if patients are able to tolerate conversion to maintenance immunosuppression with sirolimus and low-dose mycophenolate mofetil, this, too would lower exposure to potentially toxic side effects and further reduce cost and inconvenience since management of both tacrolimus and sirolimus require routine blood samples for trough levels. The added risk of another immunosuppressive agent is acceptable since all of these patients require immunosuppression to prevent loss of their graft. Adding low-dose mycophenolate mofetil should provide added protection against the risk of rejection when tacrolimus is discontinued.

**b. What is the relative risk-benefit relationship of the research compared with the risk-benefit relationship of any alternatives?**

Our group recently published an interim analysis of the first 142 patients where we presented evidence that single-dose administration of rATG is safe and associated with improved early renal function when compared to conventional alternate day dosing. Our analysis was unable to detect any differences between groups in relation to rATG-related adverse events, patient and graft survival, acute rejection, or chronic allograft nephropathy rate at six months. However, we did se clear evidence for significantly improved graft function in the group receiving the single rATG dose. A copy of the peer-reviewed published manuscript detailing these findings has been included for the board’s review. It is our belief that this dosing regimen offers the subject a favorable risk-benefit relationship when compared to conventional divided-dose administration.

**FINANCIAL OBLIGATIONS AND COMPENSATION (20-21)**

**20. Financial Obligations of the Subject**

**a. What financial obligations will the subject incur as a result of participating in the study?**

The subject (or subject’s third party payer) will be responsible for all standard costs incurred by receiving a kidney transplant. This includes the cost of the rATG, tacrolimus, sirolimus, and mycophenolate mofetil, the cost of surveillance biopsies at 6 and 12 months, and all standard lab testing and procedures performed following kidney transplantation. There will be no charge to the subject for additional blood testing that may be done as part of this protocol or the 24-month biopsy since it is not a part of standard care at this center.

**b. Will financial obligations of the subject be increased as a result of procedures performed solely for research purposes?**

All standard of care treatment will be the responsibility of the participant. Kidney transplant recipients require periodic visits and blood testing as part of standard treatment. Participants in this study may have more frequent visits because of their enrollment in the study. A kidney biopsy at 24 months is not considered standard care at this institution. Funding has been sought to cover the cost of the research-related procedure and the subject/subject’s third party payer will not be charged.

**21.** **Financial Compensation to the Subject for Participation**

**Will the subject receive any financial compensation for participation?**

Subjects will not receive any financial compensation for participation.

**POTENTIAL CONFLICTS AND PRIOR REVIEW (22-23)**

**22.** **Potential Financial Conflict of Interest**

**Does any investigator, participating physician or health care personnel (or any immediate family member [spouse, child, or sibling] of above) have a potential financial conflict of interest as defined by UNMC or University of Nebraska Board of Regents policy?**

No known potential financial conflicts of interest exists with the investigators, secondary investigators, participating physicians, health care personnel, data and administrative personnel, and/or any family members of the previously mentioned individuals.

**23. Prior IRB Review**

**a. Has this study (or one substantially similar) been previously submitted to the UNMC IRB and then withdrawn by the investigator for any other reason?** No, this study or one substantially similar has not been previously submitted to the UNMC IRB and then either withdrawn by the investigator or denied approval by the board.

**b. To the best of your knowledge, has this study (or one substantially similar) been considered by another IRB and not granted approval?**

To our knowledge, neither this study nor one similar has been considered and/or denied approval by another IRB.

**SUBJECT IDENTIFICATION, RECRUITMENT AND CONSENT/ASSENT (24-30)**

**24. Method of Subject Identification and Recruitment**

**a. How will prospective subjects be identified (e.g., from clinic population or hospital inpatient units, previous research participants, databases)?** Prospective subjects will be identified through the kidney transplant waiting list at The Nebraska Medical Center.

**b. Does the principal or secondary investigator have ethical or professional access to the names of potential subjects?** Yes.

**c. What efforts will be made to achieve appropriate study population diversity?** There are no gender, racial or ethnic eligibility criteria for this study. The absence of these limitations allows for an appropriate study population, which would reflect the available clinic population.

**d. How will prospective subjects be contacted for recruitment into the study?** Prospective study patients will be informed of the study by the investigator during a clinic visit. An introductory letter and a copy of the consent will be mailed to the patient. Preferably, discussion of the study will take place during a clinic visit before the time of transplant. The study coordinator will also be available to go over the consent form with the subject on a point-by-point basis to ensure all questions can be satisfactorily answered.

In some cases, the patient may be called in for transplant with a cadaveric donor before there has been a scheduled clinic visit during which the study could be discussed. Under this circumstance, there is there is approximately two to four hours from the time the patient arrives until the surgery begins, during which the subject and an investigator discuss the study.

**25. Process of Informed Consent**

**a. Describe the process of obtaining informed consent from the prospective subject.**

Each prospective study patient will be informed of the study by the investigator. An introductory letter and a copy of the consent will be mailed to all of the patients currently on the kidney transplant waiting list. Preferably, discussion of the study will take place during a clinic visit before the time of transplant. The study coordinator will also be available to go over the consent form with the subject on a point-by-point basis to ensure all questions can be satisfactorily answered.

In some cases, the patient may be called in for transplant with a cadaveric donor before there has been a scheduled clinic visit during which the study could be discussed. In these cases, there is usually two to four hours from the time the patient arrives until the surgery begins. When this is the case, the investigator will explain the study to the patient and any family members/friends who are present. The investigator will provide the patient with a copy of the informed consent form for their review. Time will be provided for the subject to discuss his/her participation with other family members. The investigator will then return to discuss the study further with the patient, to answer questions, and to determine whether the patient understands all the elements of the informed consent. Each subject must give signed, informed consent before treatment can begin. No patients will be enrolled without first signing the IRB-approved informed consent form.

In the past, addendums to consent have been used to inform current subjects of changes to the protocol. The preferred method to present these changes is at a subject’s routine follow-up clinic visit. If the subject is not scheduled to return to clinic in the near future, the Addendum and an invitational letter will be mailed to the subject. This will be followed by a telephone contact with the subject to answer any questions. The subject will need to sign the addendum consent before any of the additional procedures can be performed.

**b. What is the location where informed consent will be negotiated and how is the environment conducive to discussion and thoughtful consideration?**

In most cases, consent will be negotiated in the Solid Organ Clinic, located on the second floor on the Lied Transplant Center, where routine pre- and post-transplant clinics occur. Privacy and freedom from distractions in this location are conducive to both discussion and thoughtful consideration.

**c. How much time will be allotted to the process of consent?**

The process should take as much time as needed, based on understanding by the study enrollee. The average time expected is thirty minutes per subject.

- 1. **Will a delayed consent procedure be used?** Yes.
  2. **If children or adolescents will be subjects of the research, respond to the following.** Not applicable, children and/or adolescents will not be enrolled in this study.

**f. Will there be a formal process of *ongoing* reconsent (over and above reconsent associated with changes in protocol)?**

No, there will not be a formal process of ongoing reconsent; however, since the study personnel and investigators will be involved in the routine follow up of the subject, including seeing the subject in clinic visits, there will be an ongoing relationship maintained, allowing opportunities for any questions to be addressed, and informal re-consent to occur.

**26. Subject Competency**

**a. Will all adult subjects be competent to give informed consent?**

All participants will be competent to provide informed consent.

**b. Will there be any ongoing assessment of competency to continue to participate in the research?**

While there is no formal ongoing assessment of competency, subjects will be seen by investigators and participating health care personnel at regular intervals, as is standard of care. These visits will allow for assessment of subject competency.

**27. Subject and/or Representative Comprehension**

**a. How will it be determined that the subject or subject's authorized representative understood the information presented?**

The investigator or his designee will question the participant to ensure understanding of all elements of the consent document. The participant will be asked to restate, in his or her own words, all essential elements of the proposed study.

**b. If children or adolescents will be subjects of the research, how will their comprehension be assessed?**

No children and/or adolescents will be enrolled in this study.

**c. If adults with impaired competency will provide assent to participate in the research, how will their comprehension be assessed?**

All subjects will be competent to provide informed consent. Competency will be assessed by the investigators.

**28. Information Purposely Withheld**

**Will any information be purposely withheld from the subject during the research or after completion of the research?**

No information will be purposely withheld.

**29. Consent and Assent Forms**

**List all consent and assent forms which will be used in the protocol (e.g., adult consent form, parental consent form, proxy consent form, Durable Power of Attorney, youth assent form, child assent form, screening consent form, addendum consent forms).**

English and Spanish versions of adult consent forms will be used in this protocol. An addendum to consent (Addendum III) is utilized to re-consent subjects who were enrolled in this study prior to protocol modifications inclusive of extended follow-up and 24-month kidney biopsy.

**30. Documentation of Consent and Assent**

**Identify, by name, the investigator(s) and participating physicians/health care personnel who will document obtainment of informed consent and assent from the subject or the subject's legally authorized representative (i.e., sign the consent and assent form.)**

Principal Investigator: R. Brian Stevens, MD, PhD

Secondary Investigators: Lucile E. Wrenshall, MD, PhD; Wendy Grant, MD; Jean Botha, MD; David Mercer, MD; Alan Langnas, DO; Byers Shaw, Jr., MD; Gerald Groggel, MD; Jennifer Larsen, MD; James Lane, MD; Troy J. Plumb, MD; David Mercer, MD; Michael Morris, MD; Vinaya Rao, MD; Clifford Miles, MD.

The following participating physicians/ healthcare personnel will assist with the presentation of the consent form, but will not sign the consent: Anna Kellogg, RN, BSN, MS; Susan Miller, RN; Anna McGrain MSN, APRN-BC; Sally Jane Guthmiller MPAS, PA-C; Kristin Boothby MS, APRN; Kecia Christensen, MSN, APRN; Shannon Yannone, APRN; Kris Seipel, BS, CCRC; Nicole Miller, BA.

**CLINICAL TRIAL REGISTRY (31)**

**31. Clinical Trial Registry**

**Will this study be listed in a clinical trial registry (e.g., http://ClinicalTrials.gov/)?** Yes.

**What is the name of the registry, web address and identification or study number?**

Improved Induction and Maintenance Immunosuppression in Kidney Transplantation.

http://www.clinicaltrials.gov/ct2/show/NCT00556933?term=kidney+transplant&rank=8

Study number: NCT00556933

**LITERATURE REVIEW (32)**

**32. References**

**Provide a full listing of the key references cited in the background (Section II.2). The references should clearly support the stated purpose of the study.**

1. Preville X, Flacher M, LeMauff B, Beauchard S, Davelu P, Tiollier J, and Revillard JP. Mechanisms involved in antithymocyte globulin immunosuppressive activity in a nonhuman primate model. Transplantation 2001 71(3): 460-8.

2. Agha IA et al. Short course induction immunosuppression with Thymoglobulin^®^ for renal transplant recipients. Transplantation 2002 73 (3): 473-5.

3. Swanson SJ et al. Kidney Transplantation with rabbit antithymocyte globulin induction and sirolimus monotherapy. Lancet 2002 360 (9346): 1662-4.

4. Regan JF et al. Total and active Thymoglobulin^®^ levels : effects of dose and sensitization on serum concentrations. Transpl Immunol 2001 9 (1): 29-36.

5*.* Rao V, Pirsch JD, Becker BN, Knechtle SJ. Sirolimus monotherapy following Campath-1H induction. Transplant Proc. 2003 May;35(3Suppl):128S-130S.

6. A.S. Levey, J.P.Bosch, J.B. Lewis, T. Greene, N. Rogers and D. Roth. A more accurate method to estimate glomerular filtration rate from serum creatinine: a new prediction equation. Modification of Diet in Renal Disease Study Group. Ann Intern Med 1999 Mar16; 130 (6): 461-70.

7. A.S. Levey, T. Greene, J.W. Kusek, G.L. Beck, MDRD Study Group. A simplified equation to predict glomerular filtration rate from serum creatinine (abstract) J Am Soc Nephrol 2000 Sep; 11:155A.

1. The National Kidney Foundation K/DOQI Clinical Practice Guidelines for Nutrition in Renal Failure (C. Appendices – Adult Guidelines) Appendix IX. Estimation of Glomerular Filtration Rate.

9. Stevens, RB, “Calcineurin-withdrawal in renal transplant patients in a steroid-free environment.” Invited lecture, University of Minnesota, April 19, 2006.
