# Supplementary material for: A Randomized 2x2 Factorial Clinical Trial of Renal Transplantation: Steroid-Free Maintenance Immunosuppression with Calcineurin Inhibitor Withdrawal after Six Months Associates with Improved Renal Function and Reduced Chronic Histopathology
Source: PLoS One. 2015 Oct 14;10(10):e0139247. doi: 10.1371/journal.pone.0139247 (PMC4605789; doi:10.1371/journal.pone.0139247)
Supplement: S1 Criteria — (DOCX) [file pone.0139247.s002.docx]

| **Defining Criteria for Complications** | |
| --- | --- |
| **Complication/Event** | **Defining Criteria** |
| Graft failure | Permanent return to dialysis |
| Hernia requiring repair | Transplant surgery incisional hernia repair |
| ATN/DGF | Requirement for dialysis within 7 days of transplantation |
| Lymphocele requiring drainage | Drain placement within 60 days of transplantation |
| Cancer | Any malignancy with a positive biopsy report |
| rATG reaction | Fever with any hypotension, pulmonary edema, or hypoxia |
| Ureteral complications | Intervention for stricture or leak within year of transplantation |
| BK virus nephropathy | Biopsy-confirmed |
| Serum sickness | Treatment required within 60 days of rATG induction |
| Myocardial infarction | Within 30 days of transplantation |
| Cardiac arrhythmia | Within 30 days of transplantation, not in association with MI |
| Wound complications:  Within 30 days of transplantation | Wound seroma – aseptic fluid collection  Wound Infection:  Wound cellulitis – resolving with antibiotic therapy  Subcutaneous abscess (above fascia) – drainage/exudate, positive culture, antibiotic therapy  Subfascial abscess – drainage/exudate, positive culture, antibiotic therapy  Dehiscence – requiring return surgery for wound re-closure |
