# Supplementary material for: A Randomized 2x2 Factorial Clinical Trial of Renal Transplantation: Steroid-Free Maintenance Immunosuppression with Calcineurin Inhibitor Withdrawal after Six Months Associates with Improved Renal Function and Reduced Chronic Histopathology
Source: PLoS One. 2015 Oct 14;10(10):e0139247. doi: 10.1371/journal.pone.0139247 (PMC4605789; doi:10.1371/journal.pone.0139247)
Supplement: S1 Treatment — (DOCX) [file pone.0139247.s003.docx]

| **Treatment of Acute Cellular Rejection after Renal Transplantation** | | | |
| --- | --- | --- | --- |
| Biopsy Findings | Rejection Treatment | Maintenance Immunosuppression  if below target range | Maintenance Immunosuppression  if within target range |
| Borderline/Suspicious | None unless clinically indicated  (i.e., Cr 20% above baseline) | Increase oral maintenance immunosuppression to be within target ranges; repeat biopsy after treatment | Repeat biopsy  Low risk: Increase target range  High risk: Increase target |
| Focal Mild with Lab Changes – Borderline Grade 1A-1B | MP IV pulse short or standard course |  | Low risk: Increase target rangeHigh risk: Increase target range ± steroids |
| Mild-Moderate  Grade 1B-2A | MP IV pulse standard course  or rATG 5-7 days |  | Low or High risk: Increase target range ± steroids |
| Severe or Vascular  Grade 2B-3 | rATG 7-10 day course; consider IVIG ± plasmapheresis |  | Low or High risk: Increase target range ± steroids |
| Recurrent Rejection | Per biopsy findings, may be candidate for IVIG; consider adding maintenance prednisone |  | Increase oral immunosuppression target range; convert oral maintenance to steroids |
|  |  |  |  |

**Notes**

**rATG Treatment**

Administer rATG 1.5 mg/kg IV q day x 5-10 days (depending on severity and effect of treatment).

Monitor CD-3 counts as needed to assure treatment effect (goal < 50).

Adjust rATG dose for leukopenia or thrombocytopenia as follows:

If WBC < 2.0 or platelet count < 80k – Discuss with surgeon before administering next dose

If ANC < 500, give filgrastim 5 mcg/kg sq

If pulmonary edema, worsening respiratory status, or fevers exists, discuss with MD before administering.

**Pulse Steroid Treatment**

Standard (Long) Methylprednisolone Pulse (should not exceed 300 mg per dose):

MP 3 mg/kg IV qd x 3 days

MP 2 mg/kg IV qd x 2 days (may convert to PO; check with attending or Mid-Level; determining factor is daily dose)

Pred 1 mg/kg PO qd x 2 days (give in divided doses with meals)

Pred 0.5 mg/kg PO qd x 2 days

Pred 0.25 mg/kg PO qd x 2 days

Steroid-free status to be reconsidered by transplant attendings

Short Methylprednisolone Pulse:

MP 3 mg/kg IV qd x 3 days

MP 2 mg/kg IV qd x 2 days (may convert to PO; check with attending)

Pred 1 mg/kg PO qd x 2 days

Steroid-free status to be reconsidered by transplant attendings

*Note: If allograft rejection does not respond after 3-5 doses MP; start Thymoglobulin protocol.*

Ulcer Prophylaxis:

Continue or reinstitute (ranitidine or esomeprazole) x 1 month

CMV Prophylaxis:

Resume valganciclovir, valacyclovir, or acyclovir x 2 months post-rejection treatment, with renal dose adjustments. (Same med used for CMV prophylaxis post-transplant per CMV status should be used here, unless recipient CMV status has changed.)

Post-Rejection: Repeat kidney biopsy if serum Cr does not return to baseline within 7-14 days post-treatment. Also repeat biopsy within 1 month after completion of treatment.
